# Identifying the HIV-Resistance-Related Factors and Regulatory Network via Multi-Omics Analyses

**DOI:** 10.3390/ijms252111757

**Published:** 2024-11-01

**Authors:** Xueyan Long, Gexin Liu, Xinyi Liu, Chunlin Zhang, Lei Shi, Zhenglin Zhu

**Affiliations:** School of Life Sciences, Chongqing University, No. 55 Daxuecheng South Road, Shapingba, Chongqing 401331, China; longxueyan01@stu.cqu.edu.cn (X.L.); 15123887928@163.com (G.L.); 202326021038t@stu.cqu.edu.cn (X.L.); 202026021013@stu.cqu.edu.cn (C.Z.)

**Keywords:** HIV-resistant, HIV/AIDS, transcriptome, epigenomics, host dependency factors, restriction factors

## Abstract

For research on HIV/AIDS, it is important to elucidate the complex viral–host interaction, host dependency factors (HDFs), and restriction factors. However, the regulatory network of HIV-resistance-related factors remains not well understood. Therefore, we integrated four publicly available HIV-related transcriptome datasets, along with three datasets on HIV-infection-related DNA methylation, miRNA, and ChIP-seq, to predict the factors influencing HIV resistance and infection. Our approach involved differential analysis, functional annotation, and protein–protein interaction network analysis. Through comprehensive analyses, we identified 25 potential HIV-resistance-related genes (including shared *EGF*) and 24 HIV-infection-related hub genes (including shared *JUN*). Additionally, we pinpointed five key differentially methylated genes, five crucial differentially expressed microRNAs, and five significant pathways associated with HIV resistance. We mapped the potential regulatory pathways involving these HIV-resistance-related factors. Among the predicted factors, RHOA, RAD51, GATA1, IRF4, and CXCL8 have been validated as HDFs or restriction factors. The identified factors, such as JUN, EGF, and PLEK, are potential HDFs or restriction factors. This study uncovers the gene signatures and regulatory networks associated with HIV-1 resistance, suggesting potential targets for the development of new therapies against HIV/AIDS.

## 1. Introduction

Acquired immunodeficiency syndrome (AIDS), as a pandemic disease, has led to an estimated 40.4 million deaths over the past 40 years, since the first identification of human immunodeficiency virus (HIV) [1]. HIV/AIDS remains a serious threat to human health. HIV is a retrovirus belonging to the Lentivirus genus and includes two types, HIV-1 and HIV-2. Compared to HIV-2, HIV-1 is more infectious and aggressive. HIV-1 infects immune cells, such as CD4+ T cells, macrophages, and monocytes [2], leading to the progressive collapse of the human immune system and rendering individuals more susceptible to exogenous infectious diseases, such as tuberculosis [3] and various cancers [4].

An effective treatment against AIDS is the highly active antiretroviral therapy (HAART), which jointly uses three or more antiretroviral drugs to suppress HIV replication. However, HAART cannot radically cure AIDS because the virus persists in latent reservoirs, such as resting memory CD4+ T cells. Once the treatment is stopped, the viral load will rebound rapidly. HAART also carries the risk of long-term side effects, and over time, HIV can develop resistance to certain drugs [5]. Additionally, a vaccine that can permanently and completely prevent HIV infection is still not developed [6]. Although some HIV vaccines, like RV144 [7], have been developed and can reduce the risk of HIV infection to some extent, they remain largely ineffective. The obstacles include the high mutation rate of HIV populations [8] and the limited understanding of the complex interactions between HIV and the human immune system. Therefore, elucidating the molecular interactions between HIV-1 and the host is crucial for developing new antiretroviral strategies and ultimately finding a cure for AIDS.

**Figure 1:**
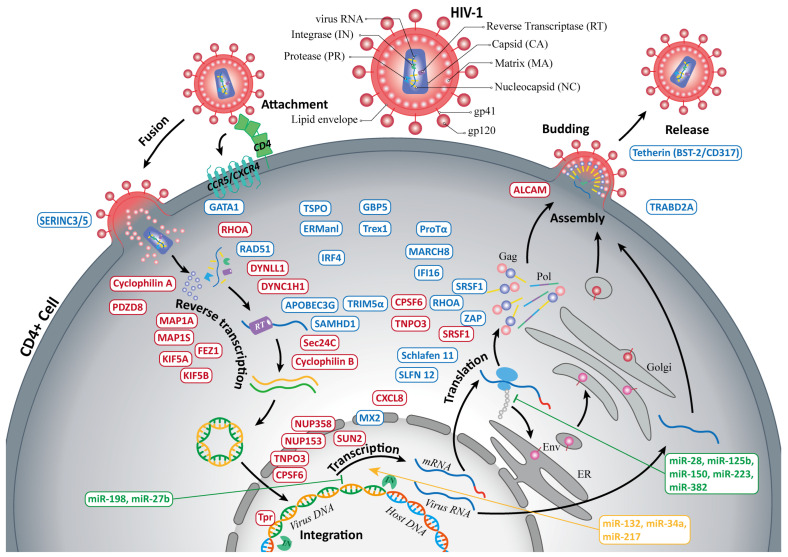
The process of HIV-1 infection of CD4+ cells and the factors that interact with HIV-1. The whole process of the HIV-1 infection cycle includes attachment, fusion, reverse transcription, integration, transcription, translation, assembly, budding, and release. Host dependency factors (shown in red) and miRNA (shown in yellow) in the host cell promote HIV-1 infection and replication, while restriction factors (shown in blue) and miRNA (shown in green) prevent it. Different elements of Gag and Pol are marked by different colors: red, matrix; dark blue, capsid; yellow, nucleocapsid; light blue, protease; purple, reverse transcriptase; green, integrase. These correspond to the colors of the proteins in the schematic diagram of the HIV-1 virus. The factors in the figure are shown schematically, and the positions of factors are not precisely associated with the location/time in the virus–host interaction. This figure is based on published research [1,9,10,11,12,13,14,15,16,17,18,19,20,21,22,23,24,25,26,27,28,29,30,31].

The progression of HIV-1 infection orchestrated by the virus’s proteins encompasses the initial and terminal stages (Figure 1). Due to the small genome size and limited number of encoded proteins, HIV-1 primarily relies on host proteins, known as host dependency factors (HDFs), to complete the entry, replication, and transmission stages of its life cycle. The HIV-1 particle comprises 15 viral proteins that interact with the host to coordinate and promote infection. These proteins include (i) four structural proteins, including matrix (MA), capsid (CA), nucleocapsid (NC), and p6, encoded by the *gag* open reading frame (ORF) and cleaved from the Gag precursor polyprotein to form the viral core; (ii) three enzymes, including protease (PR), reverse transcriptase (RT) and integrase (IN), encoded by the *pol* ORF and essential for viral replication and genome integration; (iii) an envelope polyprotein complex composed of glycoprotein 120 (gp120) and glycoprotein 41 (gp41), encoded by the *env* ORF and with a function in mediating viral entry into host cells; (iv) four accessory proteins, including virion infectivity factor (Vif), viral protein R (Vpr), viral protein U (Vpu), and negative factor (Nef), which modulate host immune responses and enhance viral replication; and (v) two regulatory proteins, including trans-activator of transcription (Tat) and regulator of expression of virion (Rev), which regulate viral gene expression and replication [9,10]. To initiate infection, HIV-1 surface glycoprotein gp120 binds to the primary cellular receptor CD4 on immune cells, including a conformational change that facilitates subsequent binding to co-receptors like C-C chemokine receptor type 5 (CCR5) or C-X-C chemokine receptor type 4(CXCR4). This binding triggers gp41-mediated fusion of HIV-1 and host cell membranes, allowing the release of the viral proteins, including CA, PR, RT, IN, p6, Vif, Vpr, Vpu, Nef, and viral RNA genome into the cytoplasm. After fusion, the capsid recruits multiple HDFs to assist in its intercellular transport, reverse transcription of viral RNA into double-stranded cDNA, nuclear entry, and integration of the viral genome into the host DNA [11,12]. The host dependency factor CPSF6 facilitates HIV nuclear entry by acting as a molecular link between CA and TNPO3. Another eight factors, including Cyclophilin A (CypA), FEZ1, MAP1A, MAP1S, PDZD8, Sec24C, NUP153, and NUP358 bind to CA. Cyclophilin B binds to Gag, while DYNLL1 and TNPO3 bind to IN. In addition, some HDFs with unknown binding sites, such as DYNC1H1, KIF5A, KIF5B, SUN2, RHOA, SRSF1, CXCL8, and Tpr, also promote multiple key stages of HIV infection [13,14,15,16]. After viral assembly, ALCAM mediates cell aggregation and promotes HIV spread [17]. Therefore, targeting HDFs, in addition to HIV-1 proteins has emerged as a potential strategy to combat HIV [11].

Concurrently, some host restriction factors also target capsid to inhibit HIV infection. In addition to HDFs, naturally occurring cellular proteins, known as restriction factors, inhibit HIV-1 replication at various stages of its life cycle through diverse mechanisms of action. While some of these restriction factors are induced by interferons (IFNs), such as IFN-α, IFN-β, and IFN-γ, others may be constitutively expressed or regulated by different pathways. Together, these factors constitute the first line of host defense against HIV-1 by recognizing and interfering with specific steps in the viral replication cycle, thus preventing infection [1,18]. To date, more than 20 restriction factors have been validated to inhibit HIV-1 and other primate lentiviruses using a variety of technological approaches, such as RNA interference, CRISPR-Cas gene editing, protein interaction analysis, and multi-omics analysis. Noted examples include the well-studied factors like APOBEC3G (antagonized by Vif), SAMHD1 (antagonized by Vpr/Vpx), Tetherin (BST-2/CD317, antagonized by Vpu), TRIM5α, MX2, ERManI, TSPO, GBP5, Trex1, ZAP, MARCH8, SERINC3/5, and Schlafen 11 [1,18,19], etc., as well as ProTα [20,21], SLFN 12 [22], TRABD2A [23], IFI16 [24], GATA1 [25], SRSF1 [26], RHOA [27], RAD51 [28], IRF4 [29], etc., which have been discovered in recent years. These restriction factors play multifunctional roles in the interactions between innate and acquired anti-HIV immunity. Understanding and searching for new restriction factors can provide new therapeutic approaches for anti-HIV replication and promote effective anti-HIV immunization. In addition, some host microRNAs (miRNAs) have been found to regulate HIV-1 replication and infectivity. miRNAs can directly interfere with the HIV-1 genome or indirectly affect infection by regulating the expression of HDFs [11]. Certain miRNAs, such as miR-28, miR-125b, miR-150, miR-223, and miR-382, can directly bind to the 3′ UTR region of HIV-1 to cause HIV-1 to enter latency [30]. The miRNAs that indirectly inhibit HIV-1 replication, such as miR-198 and miR-27b, can target cyclin T1 mRNA and suppress its protein levels [11,31]. Meanwhile, miR-132, miR-34a, and miR-217 have been reported to promote HIV-1 infection [11]. Therefore, developing miRNA mimics and inhibitors holds potential for inhibiting HIV-1 replication, infectivity, and pathogenicity [31].

In the global spread of HIV, some individuals exhibit natural resistance to the virus. Notably, the CCR5-Δ32 mutant allele is present at a high frequency (~0.10) in Caucasian populations, whereas it is virtually absent in Asian, Middle Eastern, and American Indian populations. Homozygous individuals for CCR5-Δ32 demonstrate significant resistance to HIV-1 infection, while heterozygous individuals exhibit a heightened resistance compared to wild-type individuals [32,33,34]. Additionally, in Kenya and Africa, some sex workers have been identified as naturally resistant to HIV infection [35,36]. Comparative proteome analysis between HIV-resistant individuals and HIV-negative controls has identified several anti-proteases expressed at higher levels in HIV-1-resistant individuals, playing a role in inhibiting the virus [37,38]. Despite these findings, the genomic data and related analyses of HIV-resistant individuals and populations have not yet been publicly reported. Understanding the molecular mechanism that enables these special individuals and populations to resist HIV-1 infection, as well as identifying potential restriction factors, is crucial for developing effective medicines and vaccines to prevent HIV infection and ultimately cure AIDS. With the development of next-generation sequencing technology, a significant amount of HIV/AIDS-relevant digital data have been generated, such as miRNA, DNA methylation, and ChIP-Seq data [39,40,41]. Integrated analysis of these various data types may yield novel insights. Differentially expressed miRNAs during HIV-1 infection could guide researchers in identifying host proteins that may be manipulated for therapeutic benefits. For instance, a re-analysis of gene expression and methylation regulation in glioblastoma identified an eight-gene signature with prognostic value for glioblastoma patients [42]. Building on previous efforts [43,44,45,46], we have collected and jointly analyzed HIV-related transcriptomic, miRNA, epigenomic, and proteomic data. Due to the complexity of the interaction between HIV and the host, this manuscript aims to predict new potential HIV-related factors, such as HDFs, restriction factors, and miRNAs, along with HIV-resistance pathways, regulatory networks, and related potential clinical biomarkers. This comprehensive approach is intended to provide a diversified perspective for developing new potential anti-HIV/AIDS therapies.

## 2. Results

### 2.1. Identification of Potential HIV-Related Genes

#### 2.1.1. Potential HIV-Resistance-Related Genes

**Figure 2:**
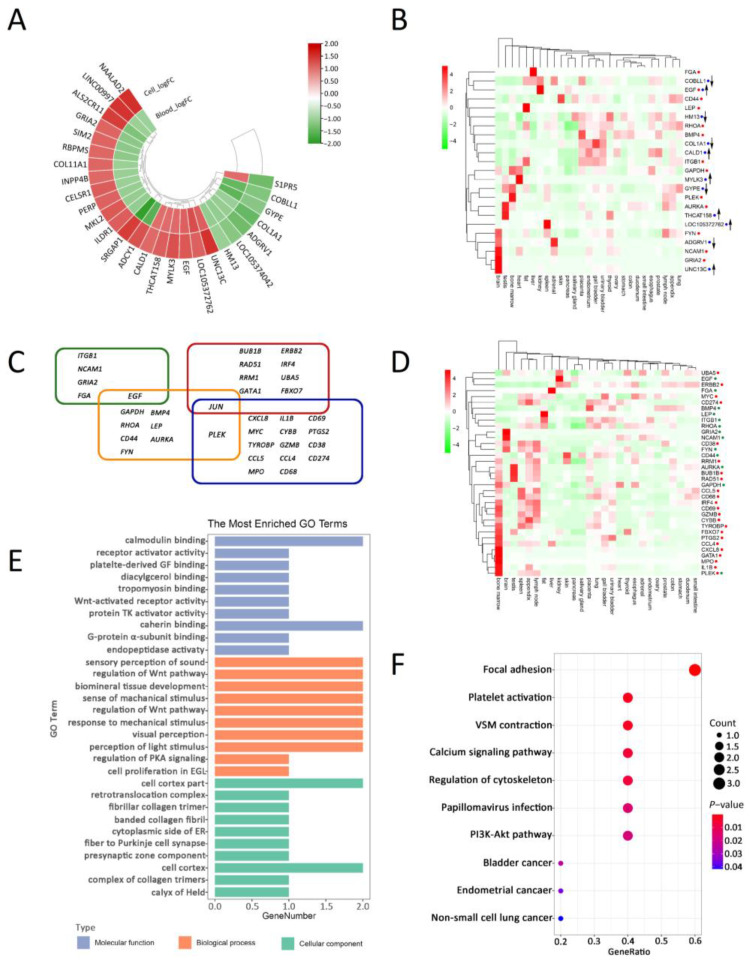
The expression and function of potential HIV-resistance-related genes. (**A**) A circular heat map shows the expression of the identified HIV-resistance-related genes in both EXP-Blood-HIV-Resistance and EXP-CD4-HIV-Resistance datasets. (**B**) A heat map displays the tissue-specific expression of the identified 25 HIV-resistance-related genes, including the 12 shared HIV-resistance-related DEGs (marked by blue cycles following gene names), and the 14 HIV-resistance-related hub genes (marked by red cycles following gene names). Up and down arrows indicate up-regulation and down-regulation, respectively. (**C**) A Venn plot of 36 hub genes across four transcriptome datasets. Hub genes from EXP-Blood-HIV-Resistance are within a green rectangle, from EXP-CD4-HIV-Resistance within an orange rectangle, from EXP-Blood-HIV-Infection within a red rectangle, and from EXP-CD4-HIV-Infection within a blue rectangle. (**D**) A heat map shows the tissue-specific expression of the 36 hub genes. Genes functioning in HIV infection are marked by red cycles following gene names, while those involved in HIV resistance are marked by green cycles. (**E**,**F**) GO enrichment analysis and pathway enrichment analysis plots of the 12 shared HIV-resistance-related DEGs, respectively.

For EXP-Blood-HIV-Resistance (GSE33580), HIV-resistant (HIV-R) samples were collected from highly exposed seronegative (HESN) populations [44]. Using RLE plots, we excluded 13 low-quality samples (Figure S1) and analyzed the remaining 73 samples with GEO2R, identifying 452 differentially expressed genes (DEGs) (81 up-regulated and 371 down-regulated) in HIV-R (Figure S2, Table S1). For EXP-CD4-HIV-Resistance (GSE14278), we discarded one low-quality sample (Figure S3) and identified 553 DEGs (279 up-regulated and 274 down-regulated) in HIV-R (Figure S4, Table S2). The intersection of DEGs from EXP-Blood-HIV-Resistance and EXP-CD4-HIV-Resistance included 27 genes (Figure 2A and Figure S5), with 12 DEGs (*COBLL1*, *GYPE*, *UNC13C*, *COL1A1*, *ADGRV1*, *LOC105374042*, *EGF*, *MYLK3*, *LOC105374042*, *THCAT158*, *HM13*, and *CALD1*) showing consistent expression patterns in both cohorts (Table S3). Analysis of these 12 DEGs in different tissues, based on a published gene expression profiling dataset [47], indicated that the brain, kidney, spleen, testis, and adrenal tissues are prominent for the expression of the 12 potential HIV-1-resistance-related genes (Figure 2B). For convenience, we refer to these as ‘the 12 shared HIV-resistance-related DEGs’ in the following description.

We identified 5 hub genes (*EGF*, *ITGB1*, *NCAM1*, *GRIA2*, and *FGA*) in the DEGs of EXP-Blood-HIV-Resistance (Figure S6) and 10 hub genes (*EGF*, *GAPDH*, *RHOA*, *JUN*, *CD44*, *FYN*, *BMP4*, *LEP*, *AURKA*, and *PLEK*) in the DEGs of EXP-CD4-HIV-Resistance (Figure S7) (Table S4). The 14 identified hub DEGs (1 shared) showed high expression in the brain, fat, bone marrow, liver, kidney, testis, and placenta (Figure 2B). Combined with the 12 shared HIV-resistance-related DEGs, we identified a total of 25 potential HIV-resistance-related genes (Table S5).

#### 2.1.2. Potential HIV-Infection-Related Genes

**Figure 3:**
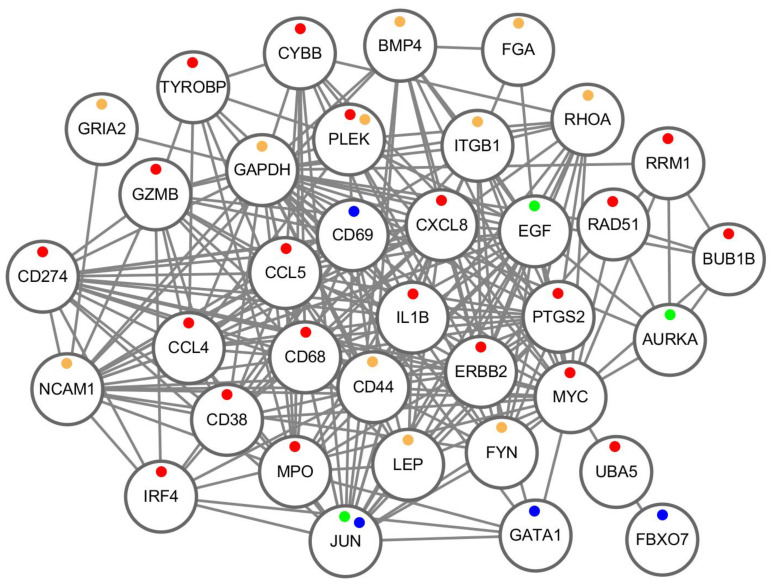
The PPI network diagram of 36 HIV-related hub genes. Red dots represent up-regulated HIV-infection-related genes, blue dots represent down-regulated HIV-infection-related genes, dark yellow dots represent down-regulated HIV-resistance-related genes and green dots represent up-regulated HIV-resistance-related genes.

To investigate the function of these potential HIV-1-resistance-related genes in response to HIV exposure, we analyzed the datasets EXP-Blood-HIV-Infection (GSE29429) and EXP-CD4-HIV-Infection (GSE73968), performing quality control for these data (Figures S8 and S9). From EXP-Blood-HIV-Infection, we identified 1458 DEGs (925 up-regulated and 533 down-regulated) (Figure S10, Table S6). From EXP-CD4-HIV-Infection, we identified 274 DEGs in naive CD4+ T cell samples (192 up-regulated and 82 down-regulated) (Figure S11), and 152 DEGs in central memory CD4+ T cell samples (119 up-regulated and 33 down-regulated) (Figure S12). In total, there were 358 DEGs (248 up-regulated and 100 down-regulated) in EXP-CD4-HIV-Infection (Table S7), with 43 up-regulated and 4 down-regulated DEGs showing consistent regulation patterns in both experiments (Table S8). Using the same method to search for HIV-R hub DEGs, we searched for HIV+ hub DEGs and identified nine hub genes (*JUN*, *BUB1B*, *RAD51*, *RRM1*, *GATA1*, *ERBB2*, *IRF4*, *UBA5*, and *FBXO7*) among the DEGs of EXP-Blood-HIV-Infection (Figure S13) and 16 hub genes (*JUN*, *PLEK*, *CXCL8*, *MYC*, *TYROBP*, *CCL5*, *MPO*, *IL1B*, *CYBB*, *GZMB*, *CCL4*, *CD68*, *CD69*, *PTGS2*, *CD38*, and *CD274*) in EXP-CD4-HIV-Infection (Figure S14). Taking together, there are 36 HIV-related hub genes (Figure 2C). We constructed a PPI network of the 36 hub genes (Figure 3) and investigated their expression in different tissues (Figure 2D). The results show that *JUN* and *PLEK* are hub genes common to both HIV resistance and HIV infection. HIV-infection-related hub genes exhibited biased high expression in bone marrow compared to HIV-resistance-related hub genes (*p*-value < 0.044, Wilcoxon test).

### 2.2. Prediction of Regulatory Networks for Potential HIV-Related Factors

Due to the lack of HIV-resistance-related epigenomic data, we utilized documented DNA-methylation and miRNA microarray data of HIV-infected samples to predict the potential regulatory network of HIV-related factors.

#### 2.2.1. Association Analysis of DMGs with HIV-Resistance-Related DEGs

We identified 13,626 differentially methylated genes (DMGs) through methylome-wide analysis using DNA-methylation data (GSE67705). Among these, 6861 genes were hyper-methylated, and 6765 genes were hypo-methylated in HIV+ patients (Table S9). We searched for the overlap between these DMGs and the HIV-R DEGs from EXP-Blood-HIV-Resistance. We found 22 up-regulated (HIV-R) and hypo-methylated (HIV+) genes (Figure S15, Table S10), and 103 down-regulated (HIV-R) and hyper-methylated (HIV+) genes (Figure S16, Table S11).

We also looked for overlaps between the DMGs and the 12 shared HIV-resistance-related DEGs, identifying two down-regulated (HIV-R) and hyper-methylated (HIV+) genes (*COBLL1*: cg20557037 and *COL1A1*: cg10820084) but no up-regulated (HIV-R) and hypo-methylated (HIV+) gene (Table S12). Furthermore, we examined the overlap between the DMGs and the 14 HIV-resistant hub genes, identifying one up-regulated (HIV-R) and hypo-methylated (HIV+) gene (*JUN*: cg15096815) and two down-regulated (HIV-R) and hyper-methylated (HIV+) genes (*GAPDH*: cg09644986 and *RHOA*: cg11409308) (Table S13). In total, we identified 125 HIV-R DEGs with DNA methylation signatures, including 103 hypo-methylated and 22 hyper-methylated genes (Tables S10 and S11). Among all identified potential HIV-resistance-related genes (Table S5), there was one up-regulated (HIV-R) and hypo-methylated (HIV+) gene and four down-regulated (HIV-R) and hyper-methylated (HIV+) genes (Table S14).

#### 2.2.2. Association Analysis of DEMs and Tat Binding Genes with HIV-Resistance-Related DEGs

We analyzed differentially expressed miRNA (DEMs) based on the microRNA data (GSE103555), identifying 47 DEMs, with 16 up-regulated and 31 down-regulated in HIV+ patients (Table S15). Target gene prediction was performed using miRDB with a prediction score > 60. We retained only those pairs where the miRNA and the predicted target gene exhibited opposite expression patterns [45]. We aimed to identify the regulation patterns of the 25 HIV-resistance-related genes using the identified DEMs, resulting in a regulatory network comprising five miRNAs and seven HIV-resistance-related genes (hsa-miR-1303: *UNC13C*, hsa-miR-4662a-5p: *ADGRV1*, hsa-miR-200a-3p: *LEP*, *PLEK* and *NCAM1*, hsa-miR-9-3p: *ITGB1* and *GRIA2*, hsa-miR-3074-5p: *NCAM1*) (Tables S16 and S17).

Additionally, we examined genes bounded by the HIV-1 Trans-Activator of Transcription protein (Tat). The analysis of ChIP-Seq data (GSE30738), which includes a genome-wide binding map of the HIV Tat protein to the human genome, revealed 5344 Tat-binding genes (Table S18), of which 124 are DEGs in EXP-Blood-HIV-Resistance (Table S19), and 4 are DEGs common to both EXP-Blood-HIV-Resistance and EXP-CD4-HIV-Resistance (Table S20).

#### 2.2.3. Functional Prediction of Potential HIV-Resistance-Related Genes

We used the R package cluster Profiler to perform gene ontology (GO) and pathway enrichment analyses (for details, see Section 4). The 452 DEGs in EXP-Blood-HIV-Resistance and the 553 DEGs in EXP-CD4-HIV-Resistance showed significant overlap. Both datasets were enriched in the function related to extracellular biological factor binding, channel activity, cell signaling transduction, and cell differentiation (Figures S17 and S18; Tables S21 and S22). They are also enriched in pathways associated with blood circulatory, neurological diseases, and cancer (Figures S19 and S20; Tables S23 and S24). DEGs in the blood or cells from HIV-infected patients exhibited functions or pathways similar to those of HIV-resistant DEGs (Figures S21–S24, Tables S25–S28). The shared 12 HIV-resistance-related DEGs are involved in functions such as focal adhesion, platelet activation, and vascular smooth muscle contraction (Figure 2E,F; Tables S29 and S30).

**Figure 4:**
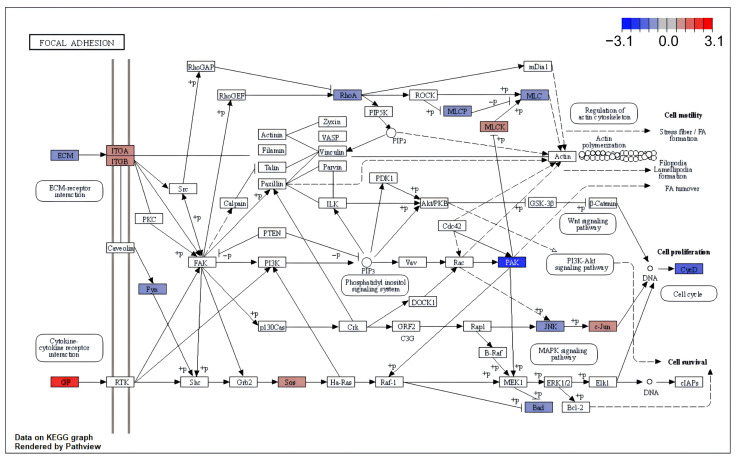
Mapped DEGs in the focal adhesion pathway, derived from a common pathway enrichment analysis based on EXP-Blood-HIV-Resistance and EXP-CD4-HIV-Resistance. Mapped DEGs are shown in colored rectangles, where red denotes up-regulation and blue denotes down-regulation. The colors of the colored boxes denote the protein expression level, corresponded to the color gradient in the upper right of the figure.

For HIV-R DEGs with different DNA methylation patterns, down-regulated HIV-R DEGs with hyper-methylation were enriched in neurological activities (Figures S25 and S26; Tables S31 and S32), while up-regulated HIV-R DEGs with hypo-methylation were enriched in the cell cycle (Figures S27 and S28; Tables S33 and S34). Tat-binding HIV-R DEGs in EXP-CD4-HIV-Resistance showed a preference for cell cycle function (Figures S29 and S30; Tables S35 and S36). In the pathway enrichment analysis of HIV-resistance-related DEGs, we identified five potential HIV-resistance-related pathways (Table S37), including focal adhesion (Figure 4), regulation of actin cytoskeleton (Figure S31), platelet activation (Figure S32), cAMP signaling (Figure S33) and gastric acid secretion (Figure S34).

#### 2.2.4. Potential HIV-Resistance Biomarker

*EGF* is the only HIV-resistance-related hub gene shared by EXP-Blood-HIV-Resistance and EXP-CD4-HIV-Resistance, and it is an up-regulated DEG in both experiments. In addition, EGF may bind to HIV-1 Tat. Therefore, we hypothesized that EGF is a potential biomarker of HIV resistance. A population survey of EGF identified 75 SNP tags (Table S38), using human population genomic data from Kenya, as the samples in EXP-Blood-HIV-Resistance and EXP-CD4-HIV-Resistance were collected from African people. By using CMap [48] to analyze the 12 shared HIV-resistance-related DEGs, we found 12 small molecules as potential therapeutic drugs for AIDS (Table S39).

#### 2.2.5. Predicted Regulatory Networks of Potential HIV-Related Factors

**Figure 5:**
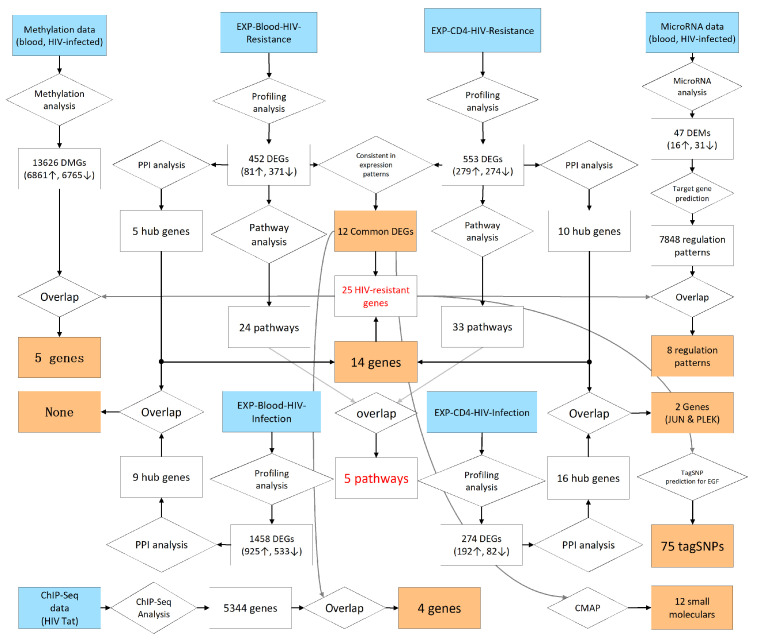
The flow chart of the entire analysis process. Blue boxes denote the start of the pipeline. Orange boxes denote the results, and diamond-shared boxes denote the analysis steps. In the rectangle boxes, the up arrow denotes up-regulation and the down arrow denotes down-regulation.

**Figure 6:**
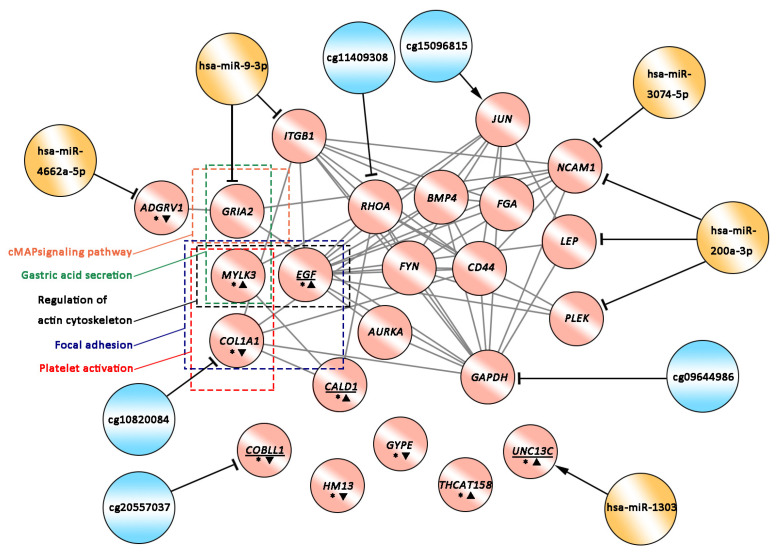
The predicted regulatory network of HIV-related factors. Red circles denote the 23 HIV-resistance-related genes (excluding two genes without annotation) based on prediction. Blue circles denote DNA methylation, and yellow circles denote DEMs. Arrows pointing to red cycles denote promotion, while lines ending with ‘T’ indicate inhibition. An asterisk (*) in red circles marks the 10 HIV-resistance-related genes with similar expression patterns in both EXP-Blood-HIV-Resistance and EXP-CD4-HIV-Resistance. Positive triangles next to “*” denote up-regulation, and inverted triangles denote down-regulation. Gene names underlined are those affected by the HIV Tat protein.

To aid understanding, we mapped the flow of comprehensive analyses for the seven datasets (Figure 5) and predicted 25 potential HIV-resistance-related genes, multiple HIV-infection-related factors (including five key DMGs and five key DEMs), and five HIV-resistance-related pathways. Based on these analyses, we constructed the regulatory network of potential HIV-related factors (Figure 6). Two genes were excluded from the regulatory network due to the absence of annotation information. In the regulatory network, most of the HIV infection-related factors appear to negatively regulate the associated genes, with only JUN hypo-methylated and hsa-miR-1303 positively regulating UNC13C. The five HIV-resistance-related pathways are mainly associated with GRIA2, MYLK3, EGF, and COL1A1.

## 3. Discussion

By integrating four HIV-related transcriptome datasets with three HIV-infection-related methylation, miRNA, and ChIP-seq datasets, we predicted a total of 25 HIV-resistance-related genes (comprising 12 DEGs and 14 hub genes, including shared *EGF* and 4 Tat-binding genes), 24 HIV-infection-related hub genes (shared *JUN*), 5 key DMGs (*COBLL1*, *COL1A1*, *JUN*, *GAPDH*, and *RHOA*), 5 key DEMs (hsa-miR-1303, hsa-miR-4662a-5p, hsa-miR-200a-3p, hsa-miR-9-3p, and hsa-miR-3074-5p), and 5 key pathways (cMAP signaling pathway, gastric acid secretion, regulation of actin cytoskeleton, focal adhesion, and platelet activation) associated with HIV resistance. The five key DEMs form eight regulatory patterns. The regulatory network of HIV-related factors offers a potential gene signature for HIV resistance, narrowing the screening range for discovering new restriction factors.

Since the identification of the first AIDS case and the isolation of HIV-1, researchers have aimed to understand the pathogenic molecular mechanisms of HIV-1 and its interactions with the host. The goal is to develop effective anti-HIV-1 therapeutic strategies from multiple angles. Although HAART can effectively reduce viral load, maintain T cell counts, and prevent opportunistic infections, viral rebound occurs once treatment is stopped, leading to reinfection [49]. Therefore, the ultimate cure for AIDS is to prevent HIV-1 from entering host cells for replication. The discovery of the CCR5-Δ32 gene mutation has brought hope for a cure, evidenced by six cases of patients being cured of HIV Five patients (“the Berlin Patient” [50], “the London Patient” [51], “the New York Patient” [52], “the City of Hope Patient” [53], and “the Düsseldorf Patient” [54]) were cured of HIV infection after receiving stem cell transplants with CCR5-Δ32 gene mutations due to fatal malignancies, and another patient showed no viral rebound post-transplant without CCR5 deletion [55]. However, for ordinary HIV-infected individuals, the risks of transplantation surgery and subsequent rejection make stem cell transplantation an impractical universal cure. Therefore, researchers continue to explore new HIV-related HDFs and restriction factors or miRNAs to find more effective therapeutic targets.

The interaction between HIV-1 and the host is complex and multifaceted. Some host proteins function as both HDFs and restriction factors, with restriction factors regulating each other and affecting their functions. Recent studies show that serine/arginine-rich splicing factor 1 (SRSF1) plays a dual role in HIV-1 replication, acting as both a restriction factor and an HDF [26]. As an HDF, SRSF1 promotes the recognition and use of splicing sites by binding to splicing regulatory elements (SREs) on HIV-1 pre-mRNA [16]. Conversely, when acting as a restriction factor, overexpression of SRSF1 significantly impairs the post-integration step of HIV-1 [26,56]. In the presence of high APOBEC3G levels, knocking down SRSF1 increases the activity of the long terminal repeat (LTR), thereby promoting overall HIV-1 replication. Additionally, our predicted HIV-resistance-related factor RHOA exhibits dual effects on HIV-1 infection. Ras homolog gene family member A (RHOA) is a small GTPase of the Rho family involved in actin cytoskeleton dynamics, cell motility, and regulation of innate immunity [57]. Multiple studies have demonstrated that RHOA can promote HIV-1 infection by enhancing receptor aggregation and virus-cell membrane fusion efficiency [15], as well as by stimulating virion production through endosomal compartments and exocytosis via its effector citron kinase (citron-K) [27]. Concurrently, RHOA inhibits HIV-1 gene expression via the NFAT binding site in the HIV LTR sequence, thus inhibiting HIV-1 replication [27,58,59].

Among the HIV-infection-related factors we identified, some have been confirmed as either HDFs or restriction factors. For example, CXCL8 is an HDF that promotes HIV-1 LTR activity by increasing the translocation of nuclear factor-κB (NF-κB) to the nucleus [14]. Restriction factors are as follows: RAD51 interacts with HIV-1 integrase (IN) and inhibits its activity [28]; the transcription factor GATA1 effectively inhibits the expression of the HIV-1 co-receptor CCR5 in human T cells and dendritic cells [25]; additionally, the significant increases in IRF4 mRNA levels are associated with T cell immune activation and represent a potential target for delaying HIV-1 infection [29]. These findings highlight the complex roles of host proteins in HIV-1 interactions. The HIV-related factors we predicted may act as both HDFs and restriction factors. Therefore, our analysis aims to identify potential gene signatures for HIV-1 resistance to facilitate the discovery of new therapeutic targets.

Looking back at our predicted regulatory network, we identified several noteworthy factors, such as RHOA, JUN, and PLEK. RHOA is already confirmed as either an HDF or restriction factor. In our analysis, *RHOA* exhibited high methylation levels and low expression (probe cg11409308). In the future, interested researchers could explore the methylation level of *RHOA* and its effects on HIV-1. *JUN* and *PLEK* are the only two hub genes common to both the EXP-CD4-HIV-Resistance and HIV infection cohorts, with no previous report linking them to HIV resistance. *JUN* is hypo-methylated, and *PLEK* is negatively regulated by hsa-miR-200a-3p. The JUN protein is a major component of the AP-1 complex, which is involved in cell growth and differentiation and promotes HIV transcription [60], suggesting JUN may act as a proviral factor. It is also known that JUN transcription is self-stimulated [61]. *PLEK* encodes Pleckstrin, the main substrate of protein kinase C (PKC), which is highly expressed in platelets and white blood cells [62,63]. PLEK also plays a role in exocytosis [64,65]. Investigating the interactions between PLEK, hsa-miR-200a-3p, and HIV-1 may provide new insights into regulatory mechanisms for HIV-1 infection.

Our results may help to establish a scientific basis for effective HIV/AIDS control strategies. However, our analysis has limitations. Firstly, due to the limited and heterogeneous datasets selected from the GEO public database, the findings might be constrained. However, the latest research has found that omics are relevant in finding treatment strategies. Secondly, the biological functions, effects, and regulatory mechanisms of the HIV-related factors predicted in this study need to be validated through basic experiments. We hope our findings will contribute to advancing HIV/AIDS treatment strategies.

## 4. Materials and Methods

### 4.1. Microarray and ChIP-Seq Data

The microarray data (GSE33580, GSE29429, GSE14278, GSE73968), miRNA data (GSE103555), DNA-methylation data (GSE67705) and ChIP-Seq data (GSE30738) were downloaded from the NCBI GEO database. The GSE33580 dataset includes blood samples from 43 HIV-resistant (HIV-R) and 43 healthy-negative control (HIV-N-C) individuals from Kenya. The GSE29429 dataset covers blood samples from 30 HIV-infected (HIV+) and 17 healthy (HIV−) individuals from Africa. The experiment was conducted on the GPL10558 platform using the Illumina HumanHT-12 V4.0 expression BeadChip. The GSE14278 dataset contains CD4+T cell samples from 9 HIV-resistant (HIV-R) and 9 healthy-negative control (HIV-N-C) individuals from Kenya, while the GSE73968 dataset includes naive CD4+ T cell samples from 3 HIV-infected (HIV+) patients and 3 healthy (HIV−) individuals, as well as central memory CD4+ T cell samples from 3 HIV-infected (HIV+) patients and 3 healthy (HIV−) individuals. The experiment was performed on the GPL6244 platform using the Affymetrix Human Gene 1.0 ST Array. Both GSE33580 and GSE14278 datasets were generated on the GPL570 platform, using Affymetrix Human Genome U133 Plus 2.0 Array. For ease of presentation, this paper refers to the GSE33580 dataset as EXP-Blood-HIV-Resistance, GSE29429 as EXP-Blood-HIV-Infection, GSE14278 as EXP-CD4-HIV-Resistance, and GSE73968 as EXP-CD4-HIV-Infection.

For miRNA data, GSE103555 (Platform GPL18058) includes samples from 10 new HIV-infected cases, 10 old HIV-infected (HIV+) cases, and 10 healthy (HIV−) controls. The GSE67705 dataset consists of DNA-methylation data from blood samples including 142 chronically HIV-infected (HIV+) patients and 44 healthy (HIV−) individuals, performed on the GPL13534 platform using the Illumina HumanMethylation450 BeadChip. GSE30738 is a ChIP-Seq dataset that presents the genome-wide binding map of the HIV-1 Trans-Activator of Transcription (Tat) to the human genome in Jurkat T cells (Jurkat-Tat cells) and involves ChIP on chip for H3K9ac in Jurkat-Tat versus Jurkat cells.

### 4.2. The Processing of Transcriptomic Microarray Data

Affy (v1.82.0) [66] was used to process the four sets of transcriptome microarray data. We performed strict quality control for microarray data using a relative log expression (RLE) plot, discarding any samples significantly different from the others [67]. After removing samples with poor data quality and performing data quality control, GEO2R (https://www.ncbi.nlm.nih.gov/geo/geo2r/, accessed on 2 November 2020) was used to analyze the differentially expressed genes (DEGs) in each set of data, with the screening criteria of *p*-value < 0.05 and |logFC| > 1. To find genes related to HIV resistance, we took the intersection DEGs (HIV-R vs. HIV-N-C) from EXP-Blood-HIV-Resistance and EXP-CD4-HIV-Resistance. To obtain genes related to HIV infection, we first took the intersection DEGs (HIV+ vs. HIV−) from naive CD4+ T cells and memory CD4+ T cells in EXP-CD4-HIV-Infection and then intersected them with the DEGs (HIV+ vs. HIV−) from EXP-Blood-HIV-Infection.

### 4.3. The Processing of DNA-Methylation and miRNA Microarray Data

Regarding DNA-methylation data, we used GEO2R to analyze differentially methylated genes (DMGs) (HIV+ vs. HIV−) with the criteria of *p*-value < 0.05 and |t| > 2. We then identified the intersection genes of down-regulated DEGs from each transcriptome dataset with hyper-methylated DMGs and the intersection genes of up-regulated DEGs with hypo-methylated DMGs. The transcriptome datasets included EXP-Blood-HIV-Resistance and EXP-CD4-HIV-Resistance.

For miRNA microarray data, we analyzed differentially expressed miRNAs (DEMs) (HIV+ vs. HIV−) using GEO2R with screening criteria of a *p*-value < 0.01 and |logFC| > 2. Subsequently, the target genes of DEMs were predicted using miRDB (https://mirdb.org/mirdb/expression.html, accessed on 3 December 2020) [68] (prediction score > 60) and analyzed alongside the DEGs from EXP-Blood-HIV-Resistance. Ultimately, this resulted in a tabulation of the predicted relationships between DEMs and DEGs.

### 4.4. The Process of ChIP-Seq Data

We used fastq-dump (v2.10.0) to convert SRA format data into fastq format and then preprocessed these fastq files using TrimGalore (v0.6.6) with requirements of a Phred quality score > 20 and reads length > 20. Quality control was performed using fastqc (v0.11.9) [69]. We mapped reads onto the human genome UCSC GRCh37/hg19 retrieved from Ensembl (https://useast.ensembl.org/index.html, accessed on 20 December 2020) [70] using bowtie2 (v2.4.2) [71] and identified ChIP-Seq enriched regions with MACS2 (v2.2.7.1) [72]. Finally, we used an R package ChIPseeker (v1.40.0) [73] for annotation. We selected correlated genes that were not only located within ChIP-Seq peaks but were also DEGs from EXP-Blood-HIV-Resistant and performed the same selection for shared DEGs from both EXP-Blood-HIV-Resistant and EXP-CD4-HIV-Resistant.

### 4.5. GO and Pathway Enrichment Analysis

We used the R package clusterProfiler (v4.12.6) [74] for Gene Ontology (GO) and Kyoto Encyclopedia of Genes and Genomes (KEGG) analysis with a cut-off of *p*-value < 0.05 and ggplot2 (v3.3.3) for visualizations. We conducted analyses for all DEGs, up-regulated DEGs with hypo-methylated DMGs, down-regulated DEGs with hyper-methylated DMGs, and ChIP-Seq correlated genes.

### 4.6. Protein–Protein Interaction Network and Hub Gene Selection

We used the Search Tool for the Retrieval of Interacting Genes (STRING) (https://cn.string-db.org/, accessed on 25 January 2021, v11.0) [75] to generate protein–protein interaction (PPI) networks and viewed the results using Cytoscape (v3.10.2) [76]. In Cytoscape, we employed cytoHubba [77] to identify hub genes using the MNC, Degree, EPC, BottleNeck, Closeness, and Radiality algorithms for taking intersections. We selected the top 5% of cases with the highest scores. Additionally, we looked for overlapping DEGs between EXP-Blood-HIV-Resistant and EXP-CD4-HIV-Resistant.

### 4.7. Other Analysis

We used Haploview (v4.2) [78] to obtain, view, and analyze the SNP information of genes. The NCBI project HPA RNA-Seq normal tissues (https://ncbi.nlm.nih.gov/bioproject/PRJEB4337/, accessed on 7 February 2021) was utilized to search for the gene expression information across different tissues. Additionally, we used the Connective Map (CMAP, https://www.broadinstitute.org/connectivity-map-cmap, accessed on 25 February 2021) to identify potential small molecular drugs based on DEGs, filtering results with a score > 0 and *p*-value < 0.01.

## 5. Conclusions

This study preliminarily identified 25 HIV-resistance-related genes and 24 HIV-infection-related hub genes. We predicted the regulatory network of HIV/AIDS resistance-related factors based on differentially methylated genes (DMGs), differentially expressed miRNAs (DEMs), and key pathways. Among the predicted factors, RHOA is a proven host dependency factor and restriction factor, while CXCL8 is a known host dependency factor. RAD51, GATA1, and IRF4 are recognized restriction factors. Additionally, JUN is a potential HDF, and EGF and PLEK are potential restriction factors. Future research can further explore the interactions between these factors and HIV. This study provides potential gene signatures for HIV resistance, offering new avenues for understanding and combating HIV/AIDS.

## Data Availability

The source data used in this study are available in the NCBI GEO database (GSE33580, GSE29429, GSE14278, GSE73968, GSE103555, GSE67705, GSE30738).

## Supplementary Materials

The supporting information can be downloaded at: https://www.mdpi.com/article/10.3390/ijms252111757/s1.
