# Supplementary material for: Identifying the HIV-Resistance-Related Factors and Regulatory Network via Multi-Omics Analyses
Source: Int J Mol Sci. 2024 Nov 1;25(21):11757. doi: 10.3390/ijms252111757 (PMC11546959; doi:10.3390/ijms252111757)
Supplement: Supplementary file 1 [file ijms-25-11757-s001.zip › Supplementary Figures.pdf]

Supplementary Materials for

Identifying the HIV-Resistance-Related Factors and Regulatory Network via Multi-Omics Analyses

Xueyan Long[†], Gexin Liu[†], Xinyi Liu, Chunlin Zhang, Lei Shi[*], Zhenglin Zhu[*]

School of Life Sciences, Chongqing University, Chongqing, China

† These authors contribute equally;

* Correspondence: shil@cqu.edu.cn (L.S.); zhuzl@cqu.edu.cn (Z.Z.); Tel.: +86-23-6512-2686(Z.Z.)

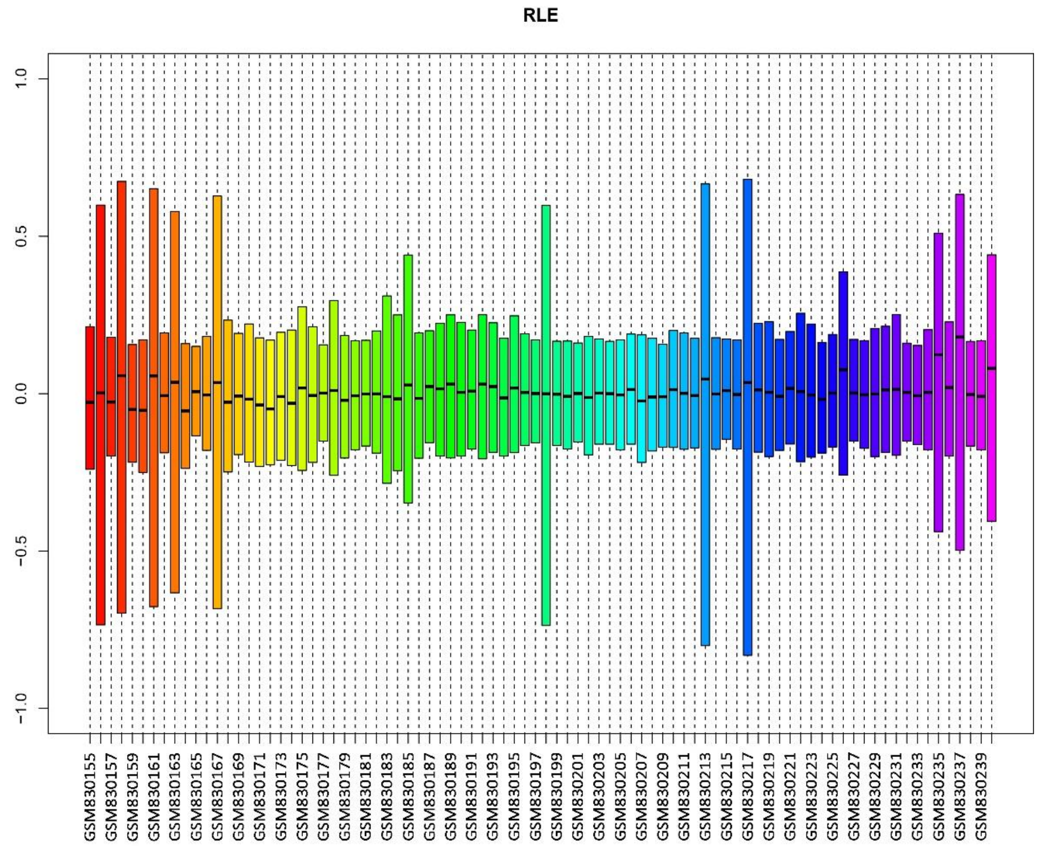

Figure S1. The RLE box plot of samples in the transcriptome data set EXP-Blood-HIV-Resistance. This figure is produced by the affy package for quality control.

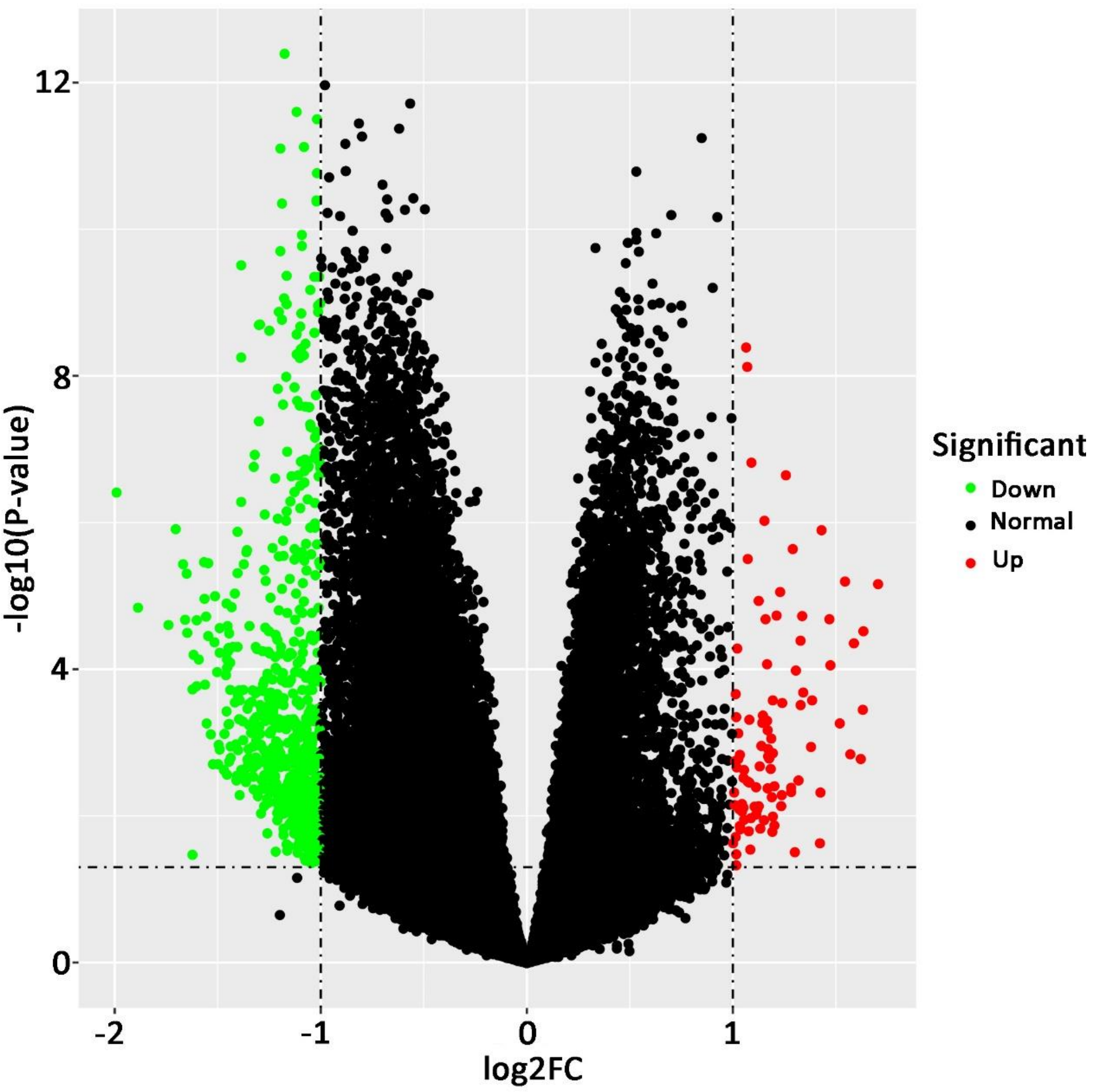

Figure S2. The volcano plot of gene expression changes between HIV-R and HIV-N-C in EXP-Blood-HIV-Resistance.

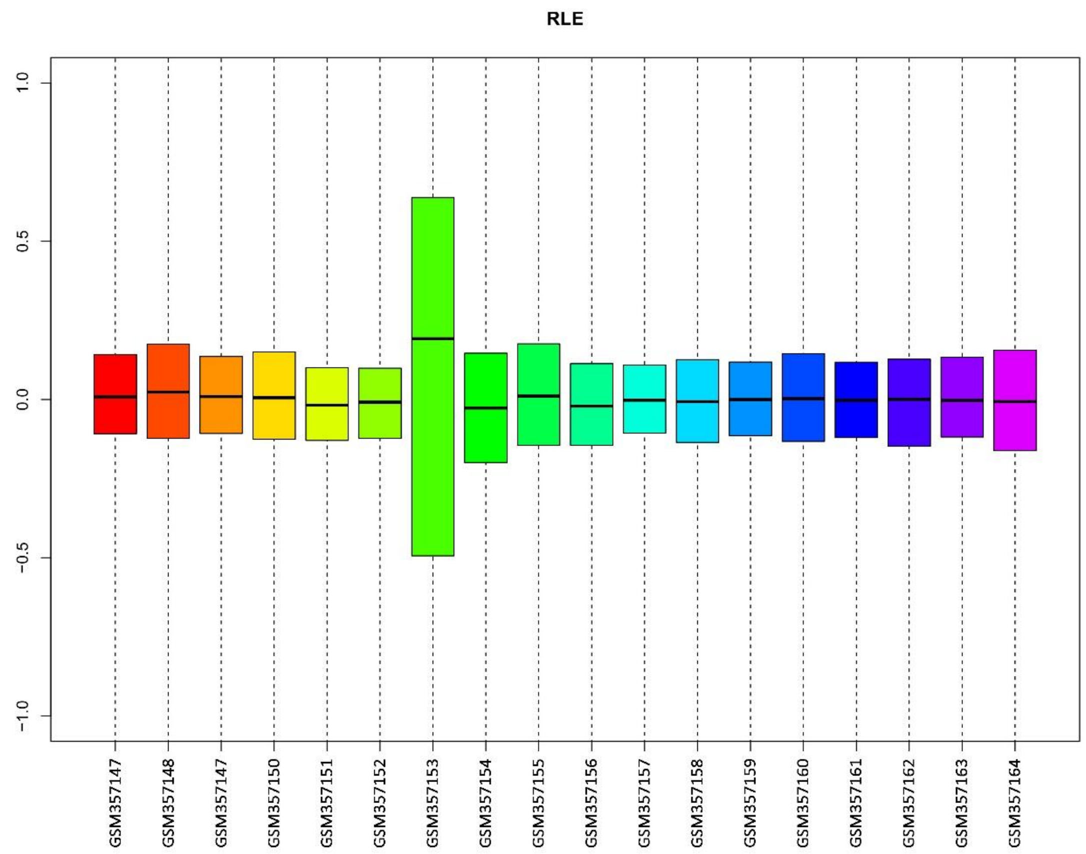

Figure S3. The RLE box plot of samples in the transcriptome data set EXP-CD4-HIV-Resistance. Other legends follow Figure S1.

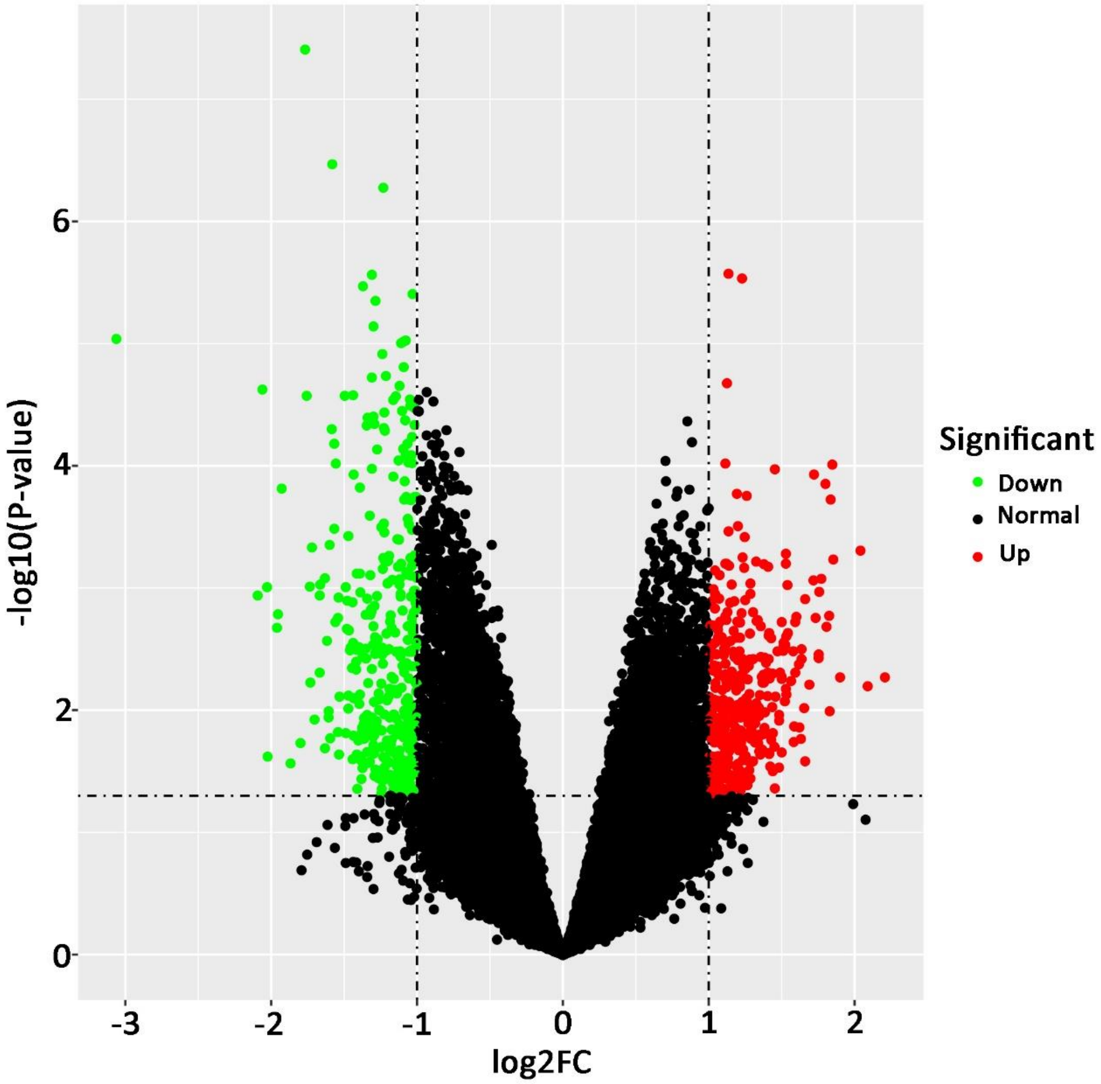

Figure S4. The volcano plot of gene expression changes between HIV-R and HIV-N-C in EXP-CD4-HIV-Resistance.

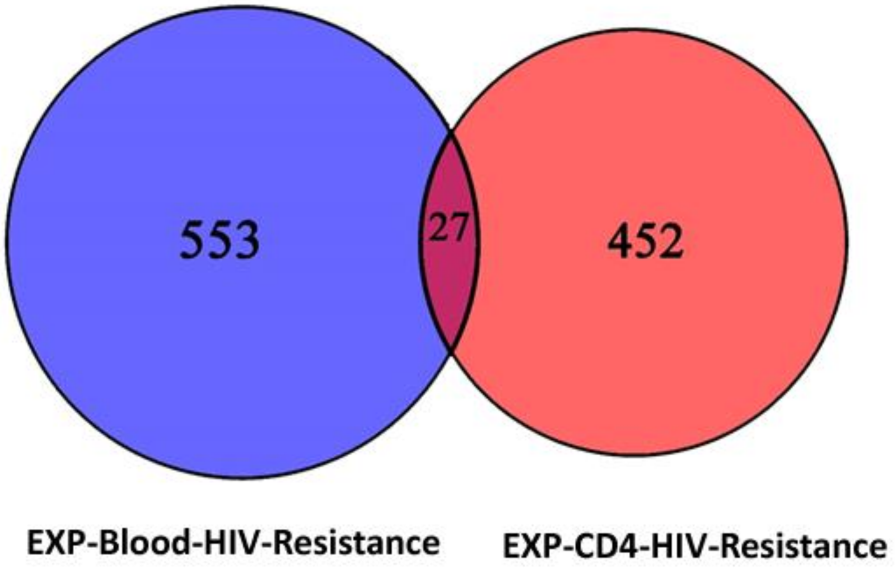

Figure S5. A Venn plot showing the overlap of DEGs from EXP-Blood-HIV-Resistance and EXP-CD4-HIV-Resistance.

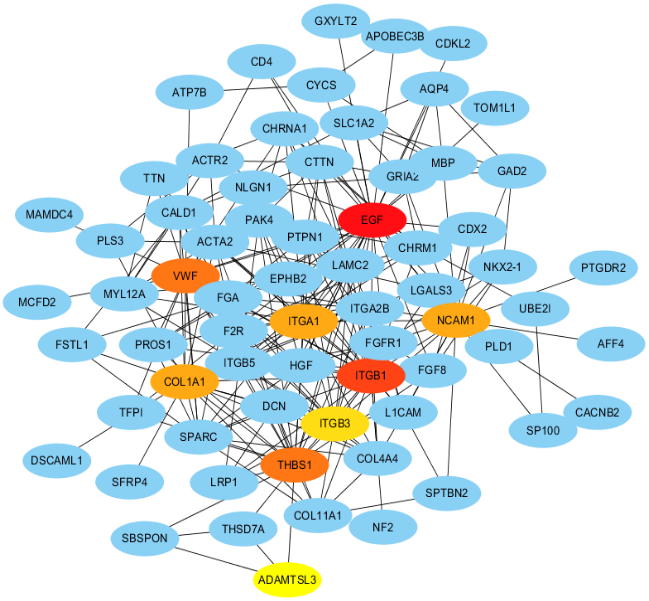

Figure S6. The PPI network of DEGs of EXP-Blood-HIV-Resistance.

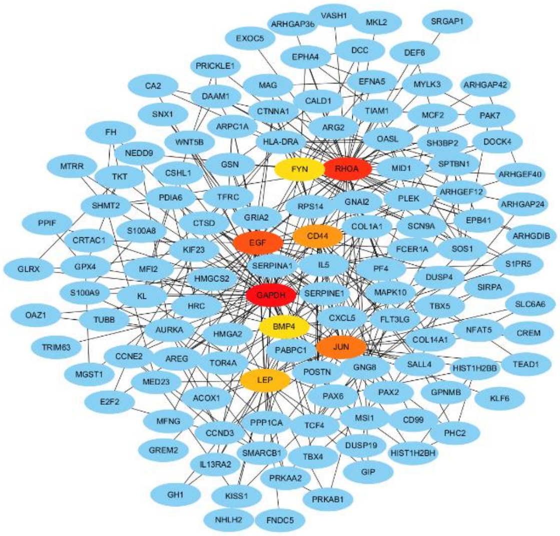

Figure S7. The PPI network of DEGs of EXP-CD4-HIV-Resistance.

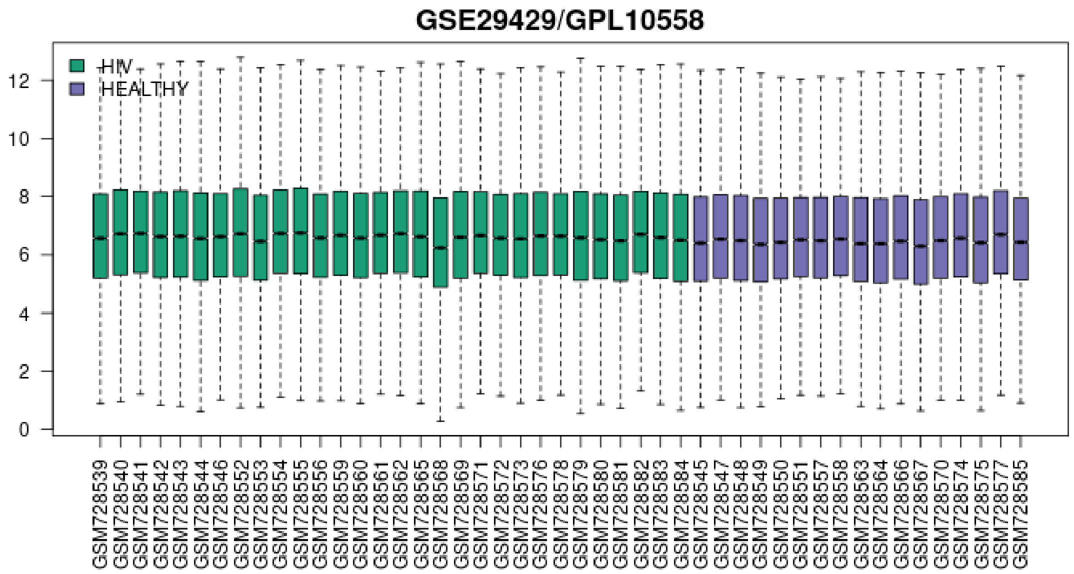

Figure S8. Box plot of the transcriptome dataset EXP-Blood-HIV-Infection. This figure is produced by GEO2R for quality control. X axis are samples. Y are median-centered values indicative if data are normalized and cross-comparable.

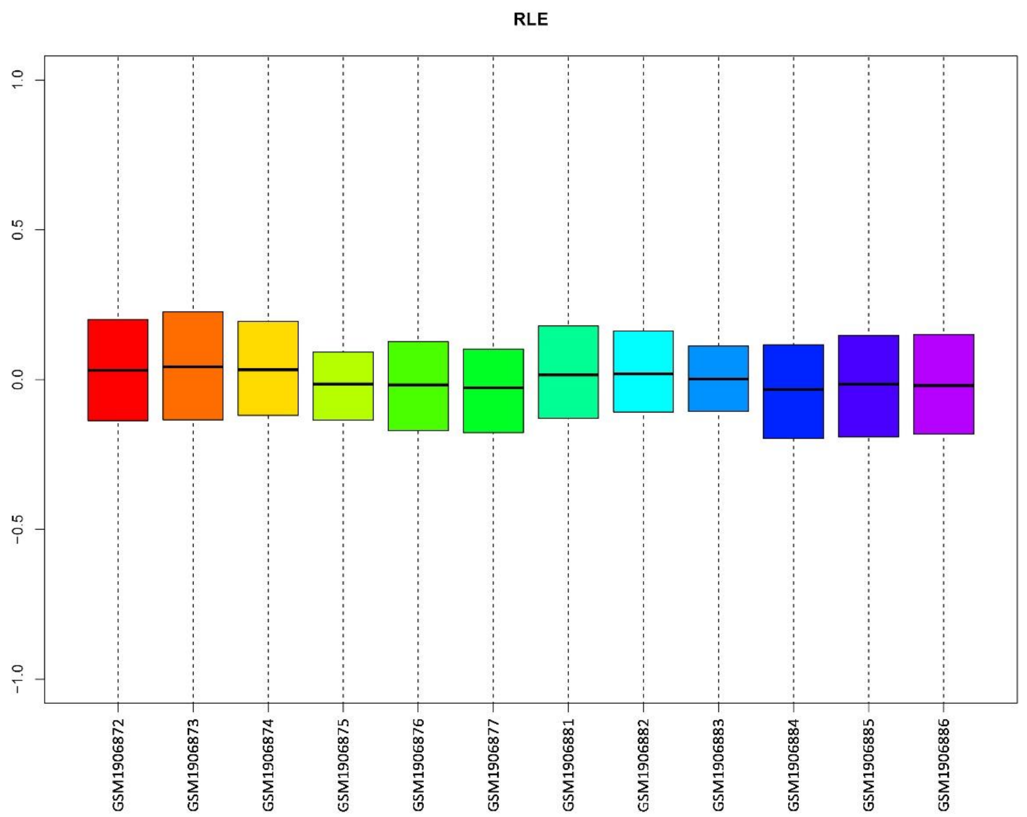

Figure S9. The RLE box plot of samples in the transcriptome data set EXP-CD4-HIV-Infection. Other legends follow Figure S1.

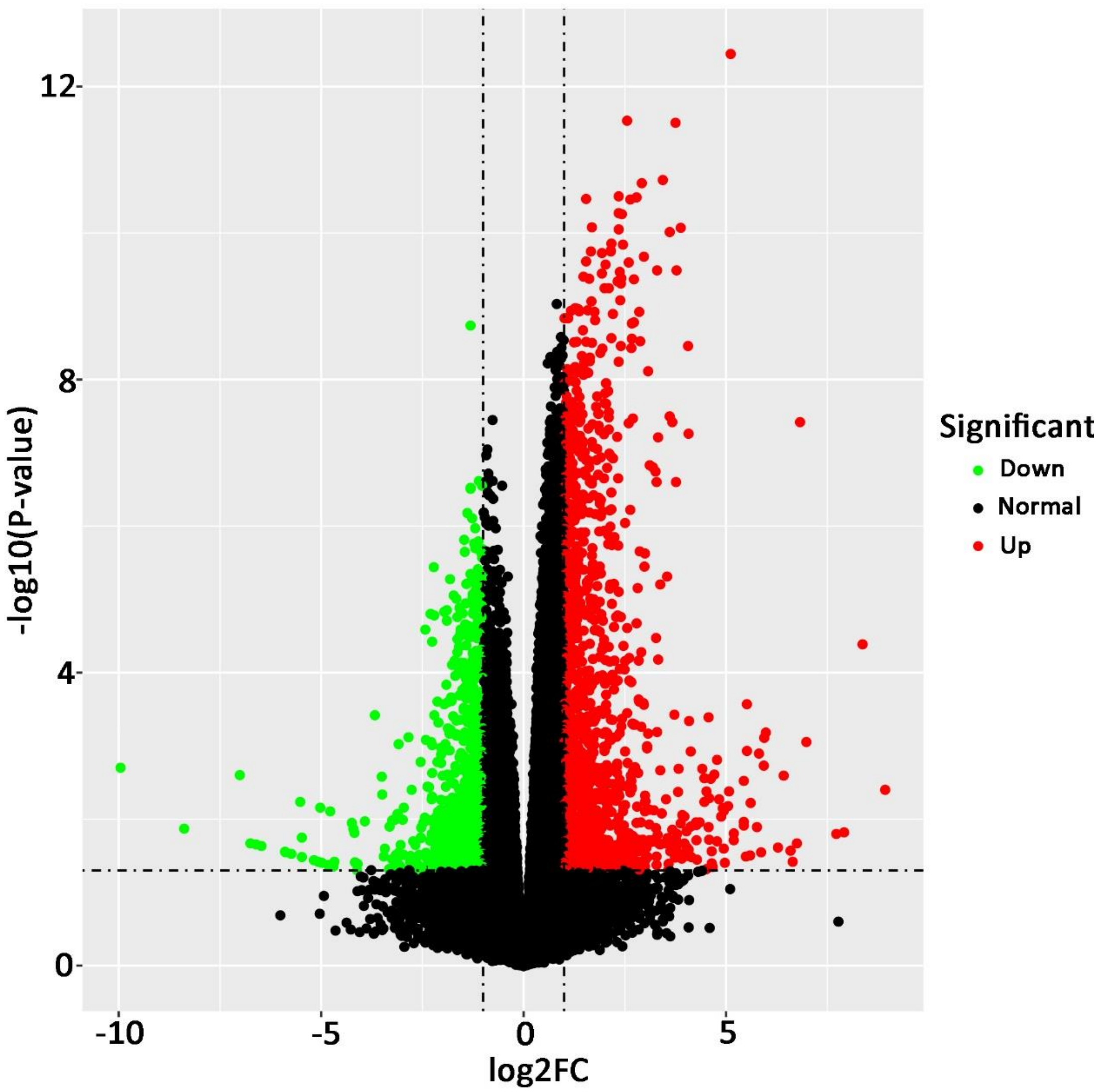

Figure S10. The volcano plot of gene expression changes between HIV+ and HIV- in EXP-Blood-HIV-Infection.

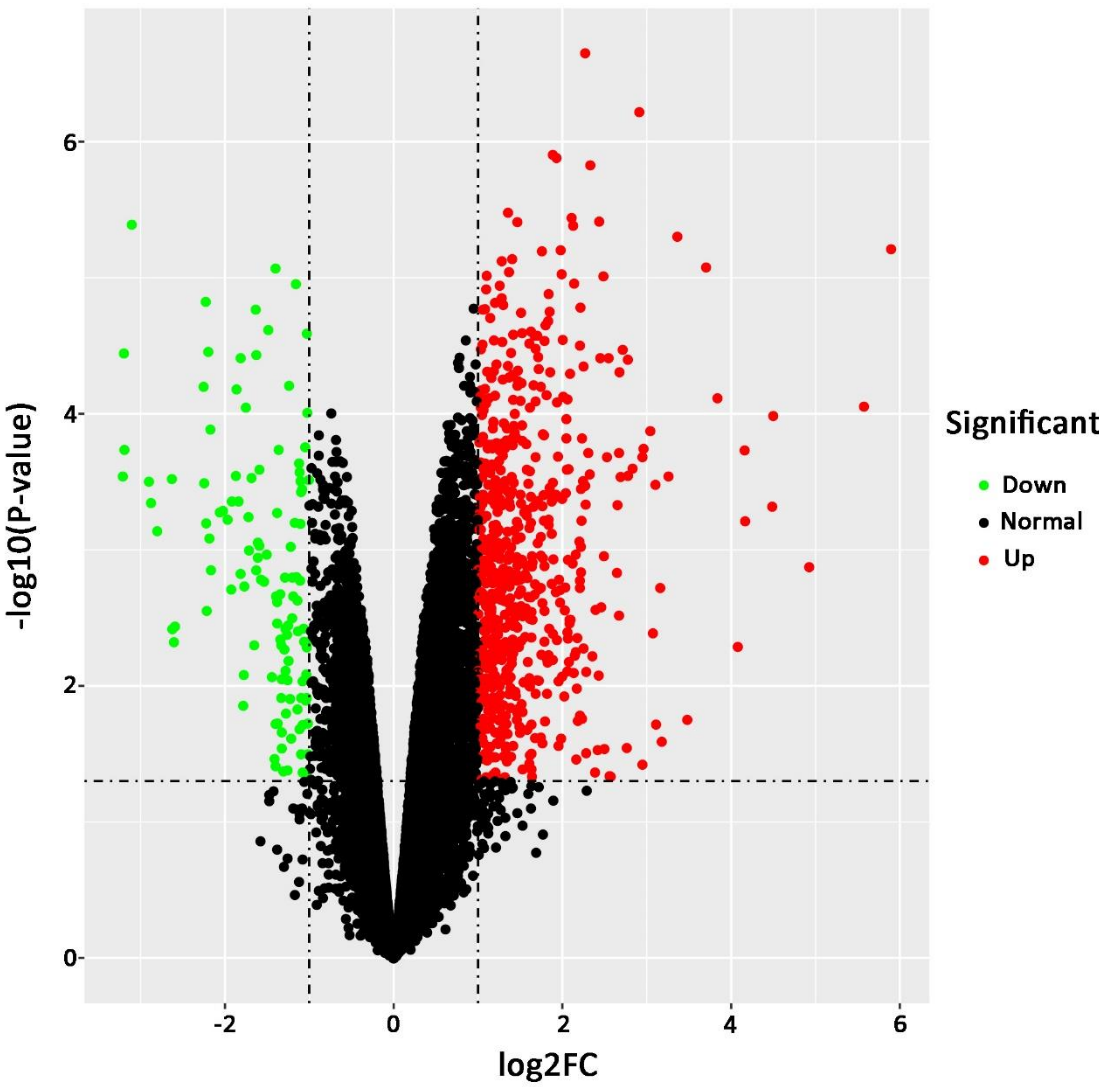

Figure S11. The volcano plot of gene expression changes between HIV+ and HIV- in EXP-Blood-HIV-Infection (naive CD4+ T cell).

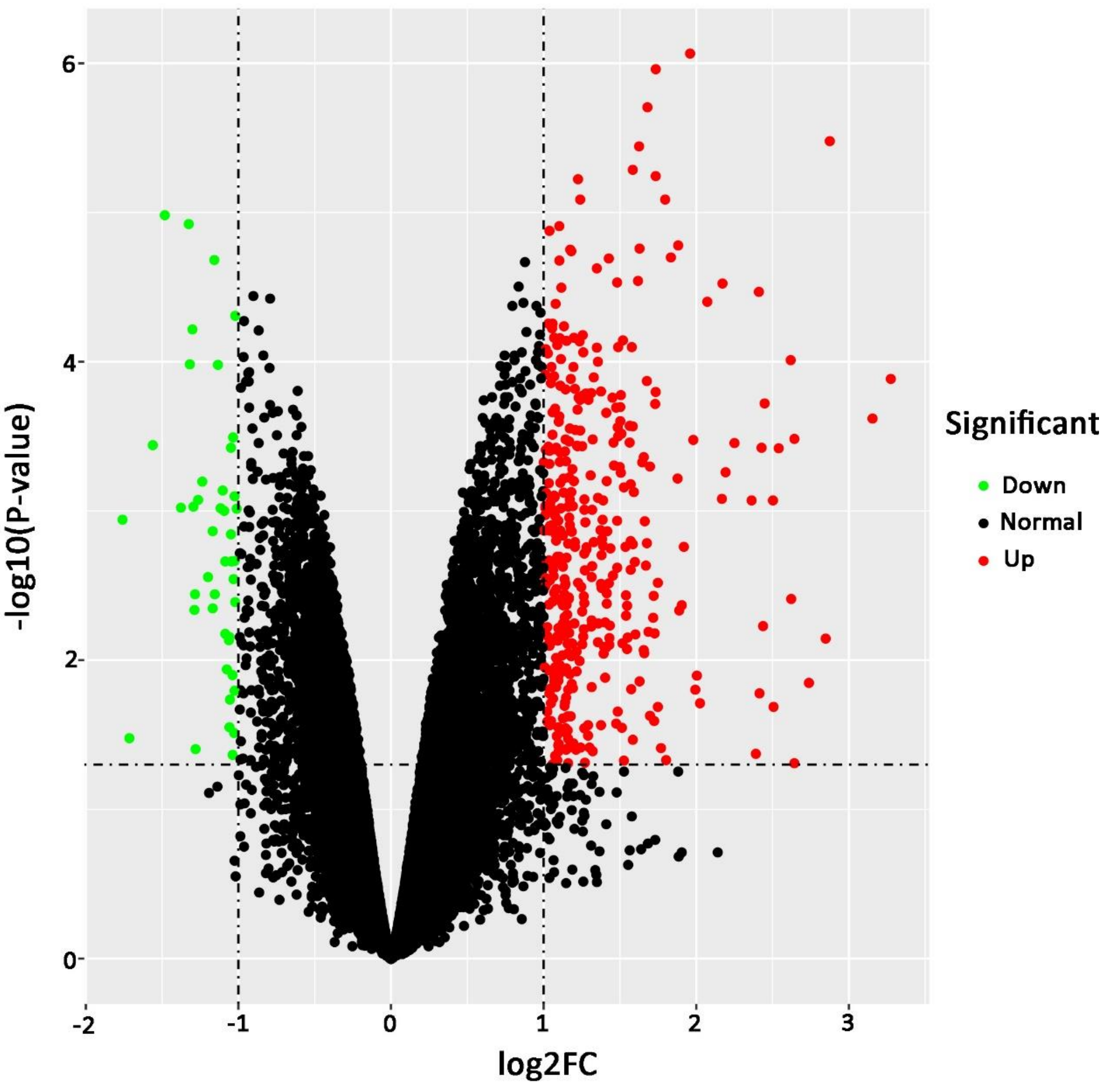

Figure S12. The volcano plot of gene expression changes between HIV+ and HIV- in EXP-Blood-HIV-Infection (central memory CD4+ T cells).

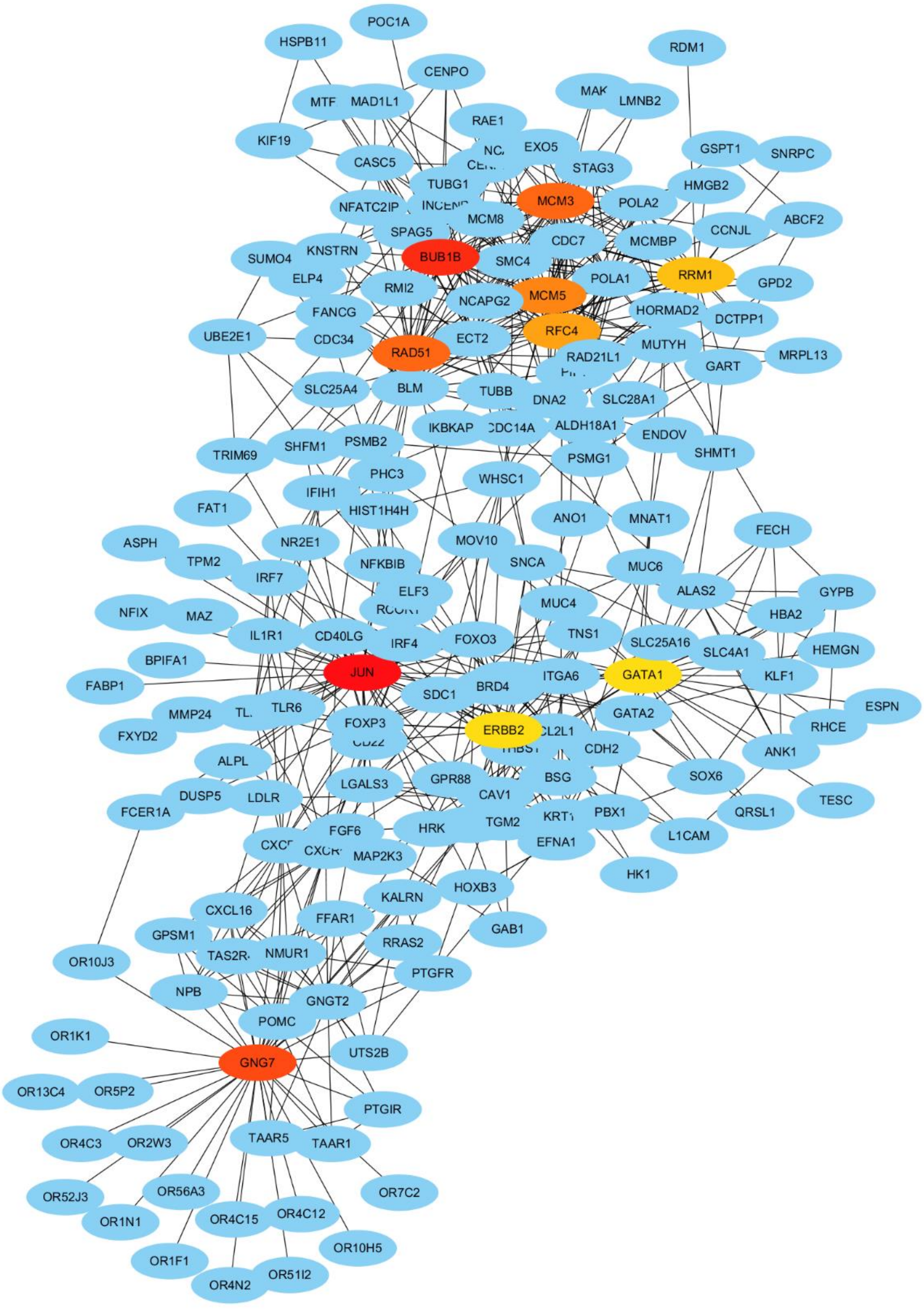

Figure S13. The PPI network of DEGs of EXP-Blood-HIV-Infection.

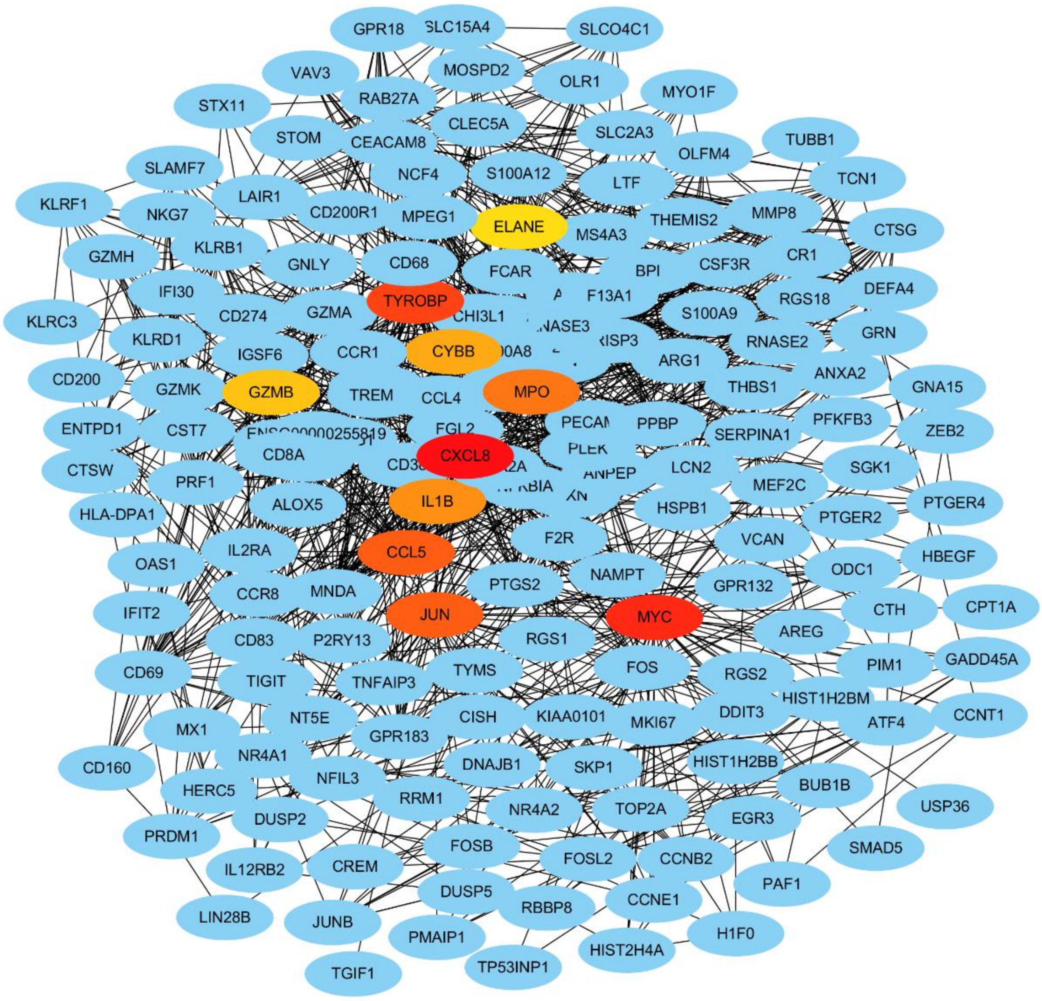

Figure S14. The PPI network of DEGs of EXP-CD4-HIV-Infection.

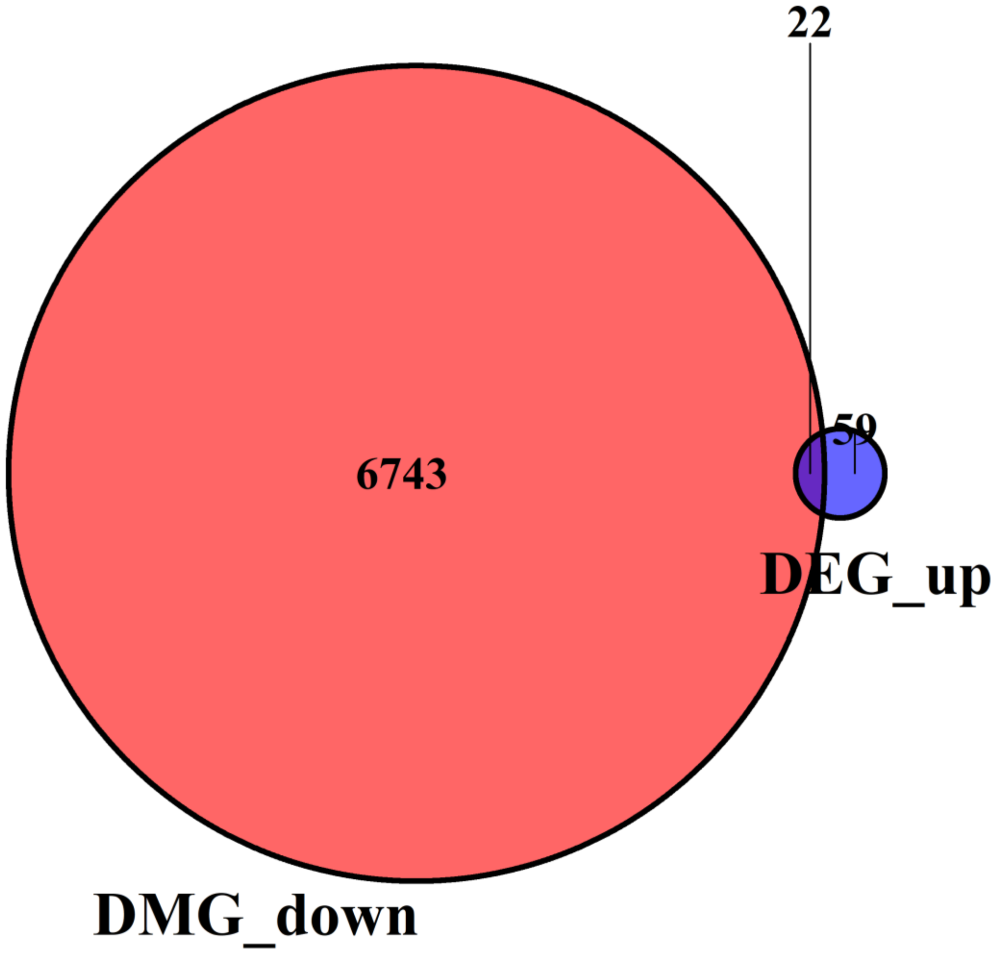

Figure S15. The Venn plot of hypo-methylated up-regulated genes. The overlap is the HIV-R up-regulated DEGs and the hypo-methylated DMGs.

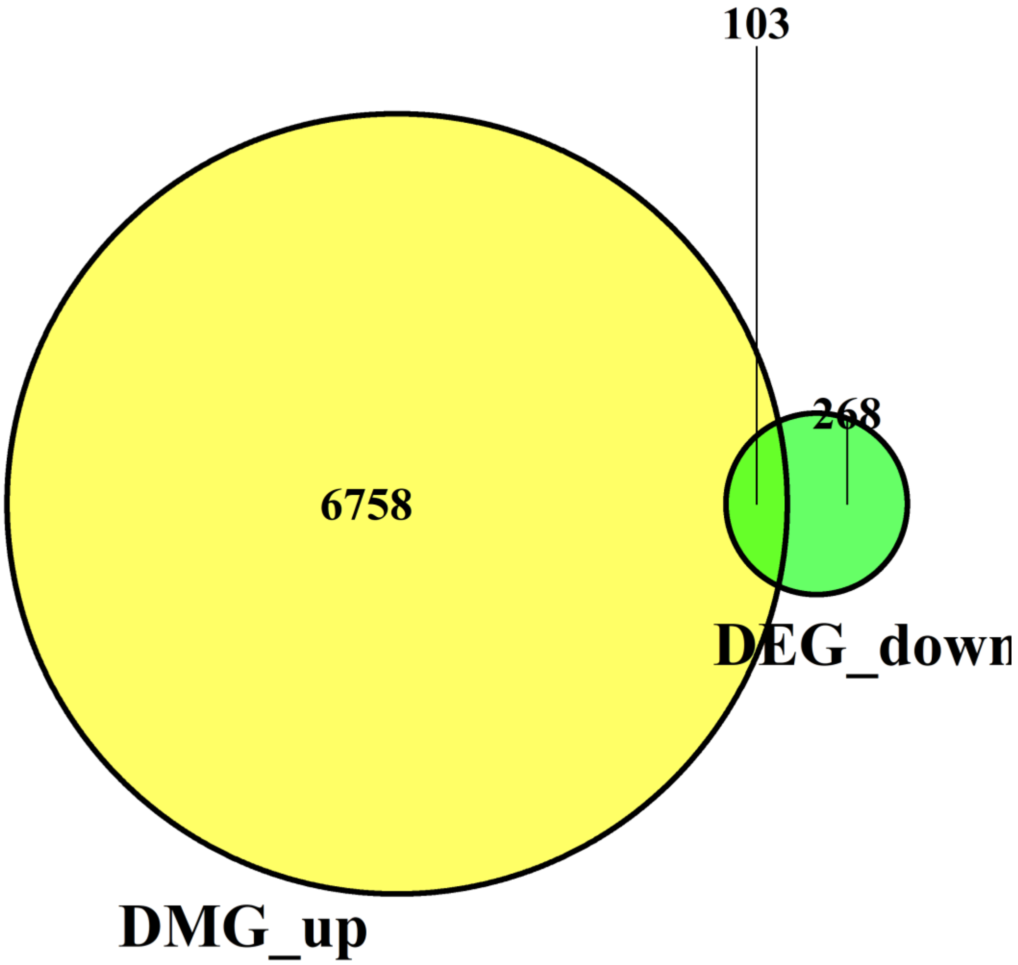

Figure S16. The Venn plot of hyper-methylated down-regulated genes. The overlap is the HIV-R down-regulated DEGs and the hyper-methylated DMGs.

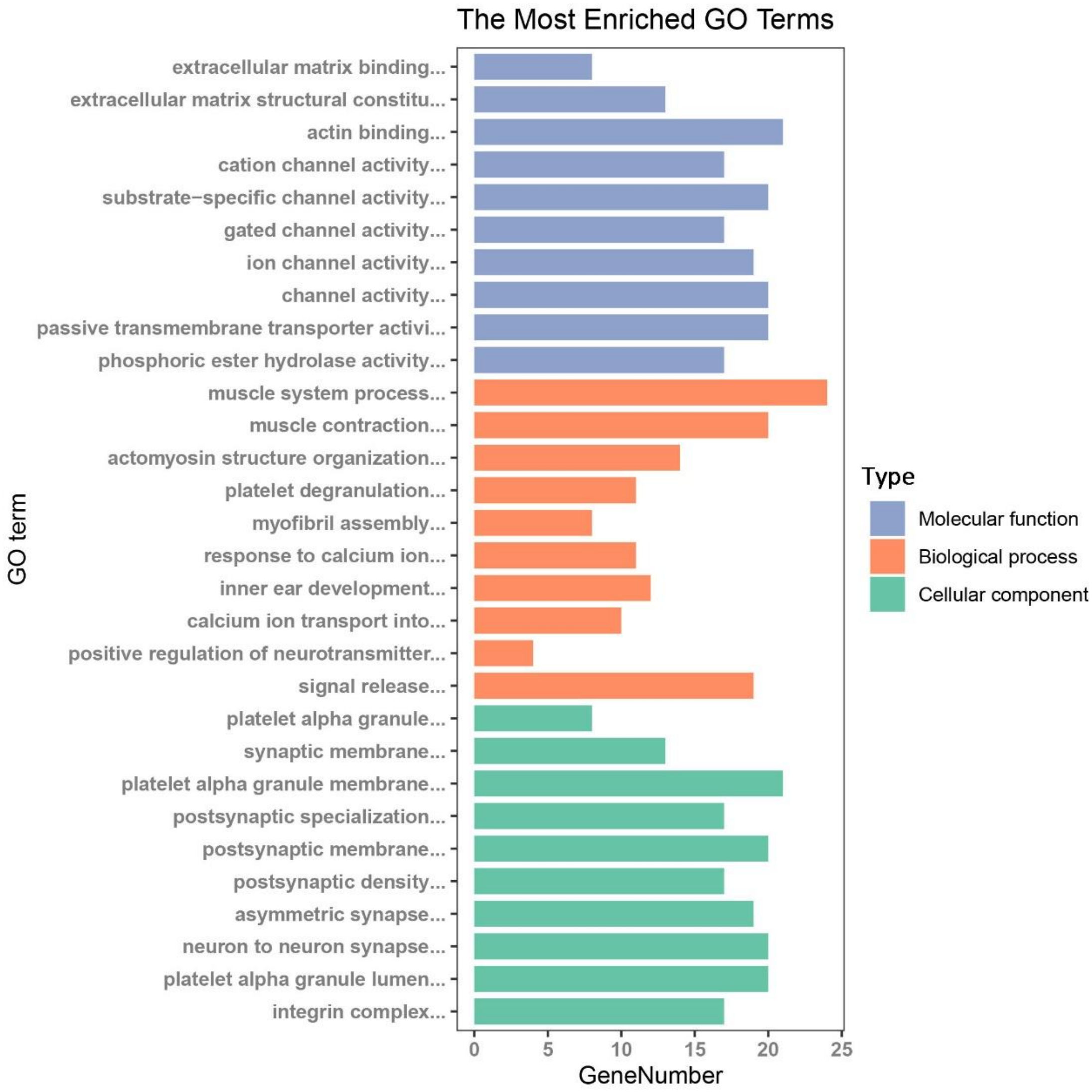

Figure S17. The GO enrichment analysis plot of DEGs in EXP-Blood-HIV-Resistance.

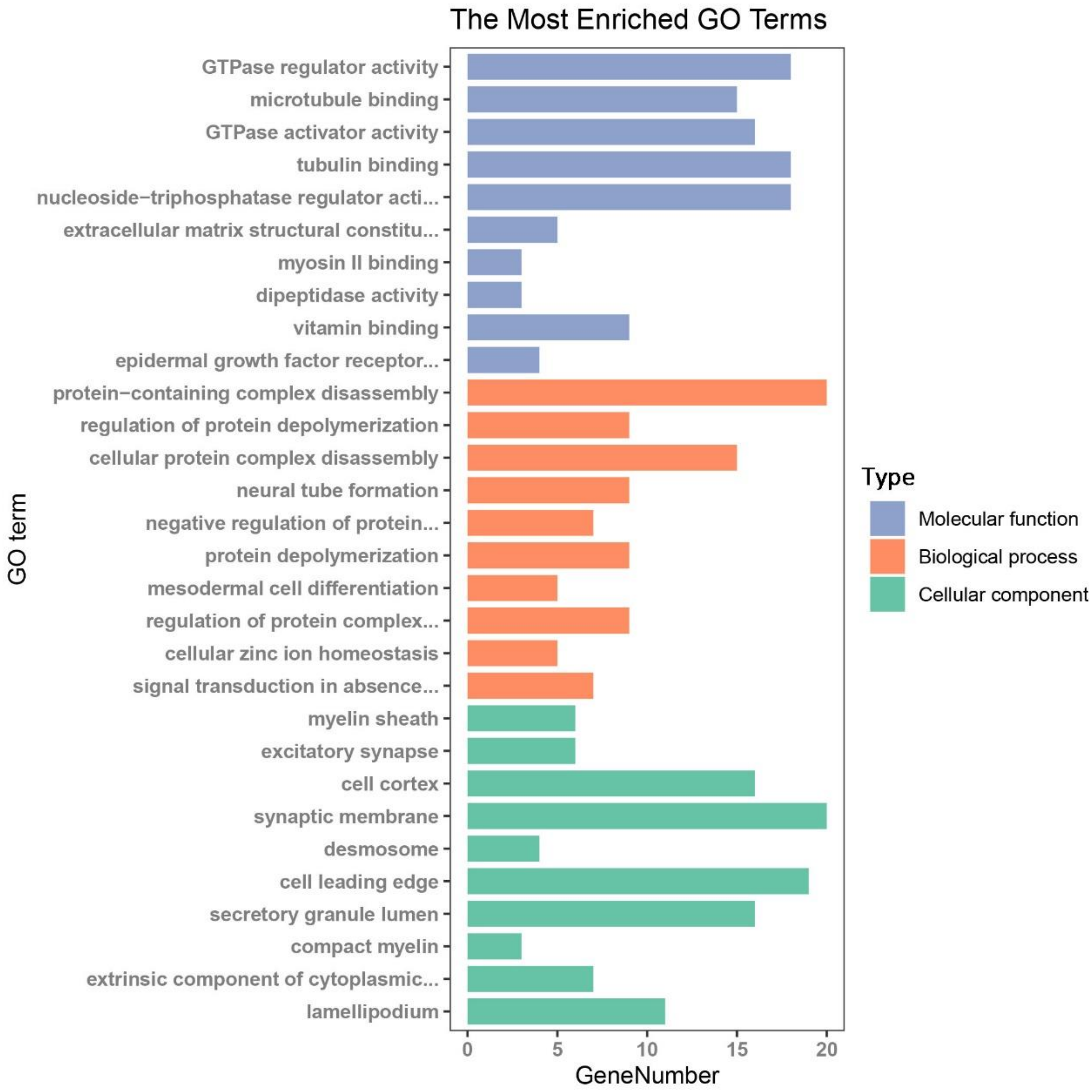

Figure S18. The GO enrichment analysis plot of DEGs in EXP-CD4-HIV-Resistance.

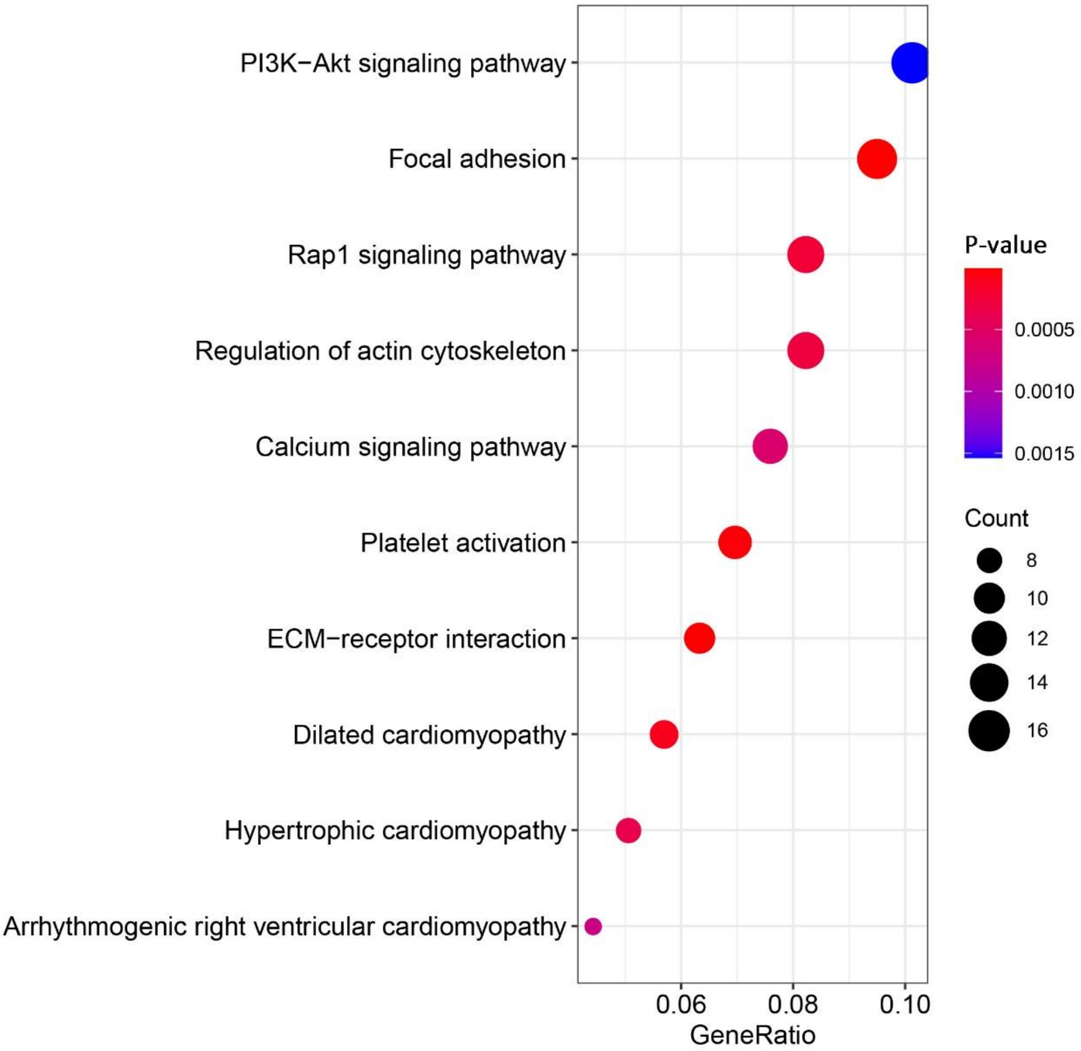

Figure S19. The pathway enrichment analysis plot of the DEGs in EXP-Blood-HIV-Resistance.

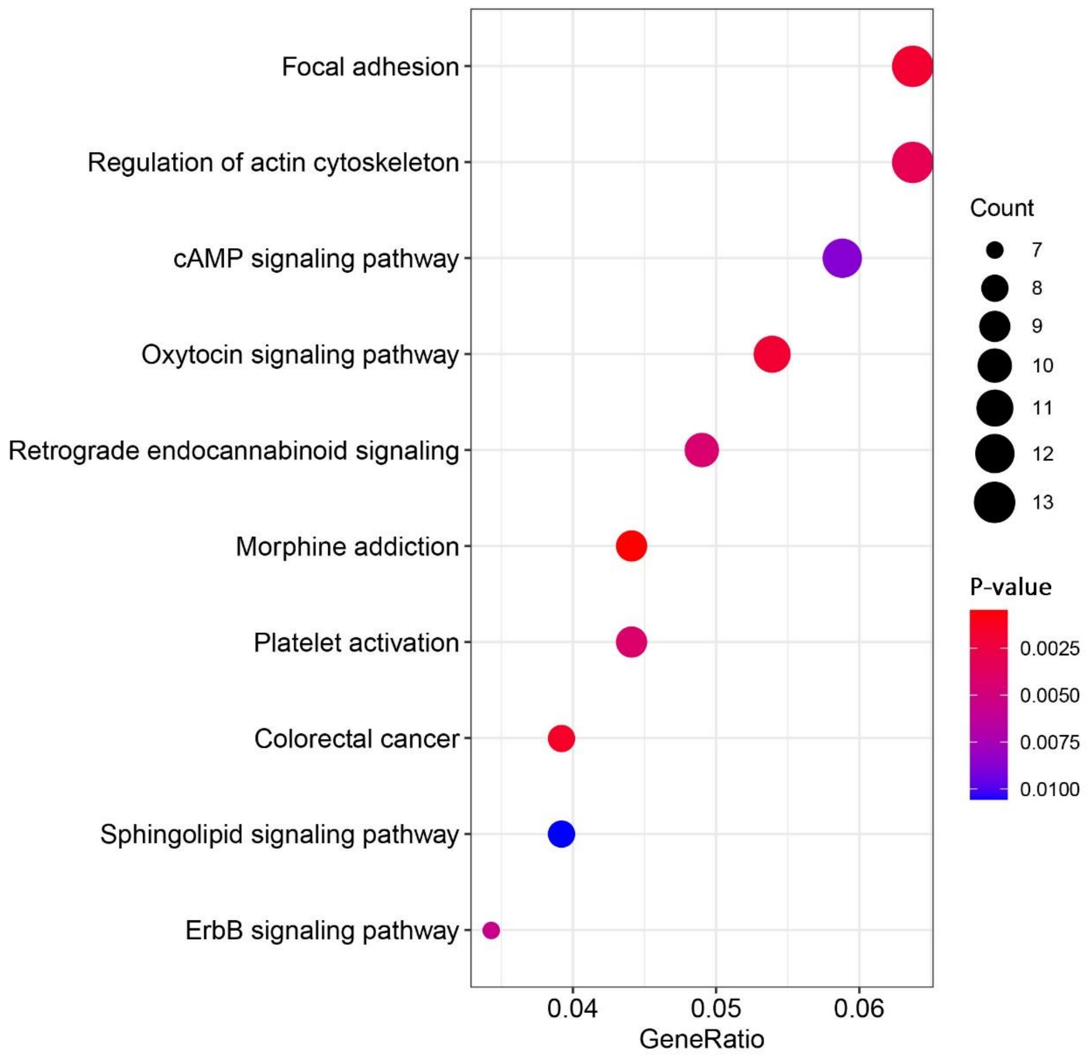

Figure S20. The pathway enrichment analysis plot of the DEGs in EXP-CD4-HIV-Resistance.

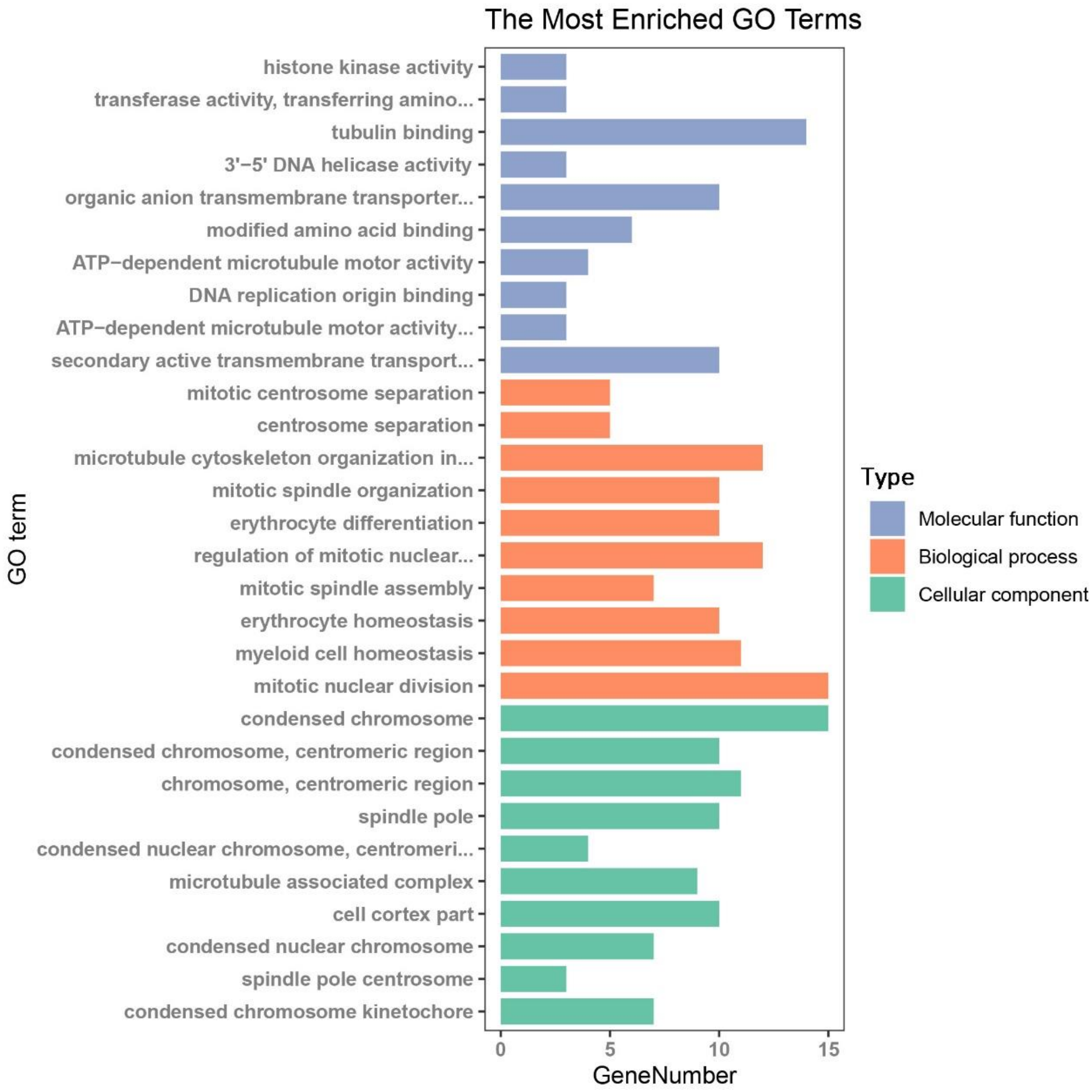

Figure S21. The GO enrichment analysis plot of the DEGs in EXP-Blood-HIV-Infection.

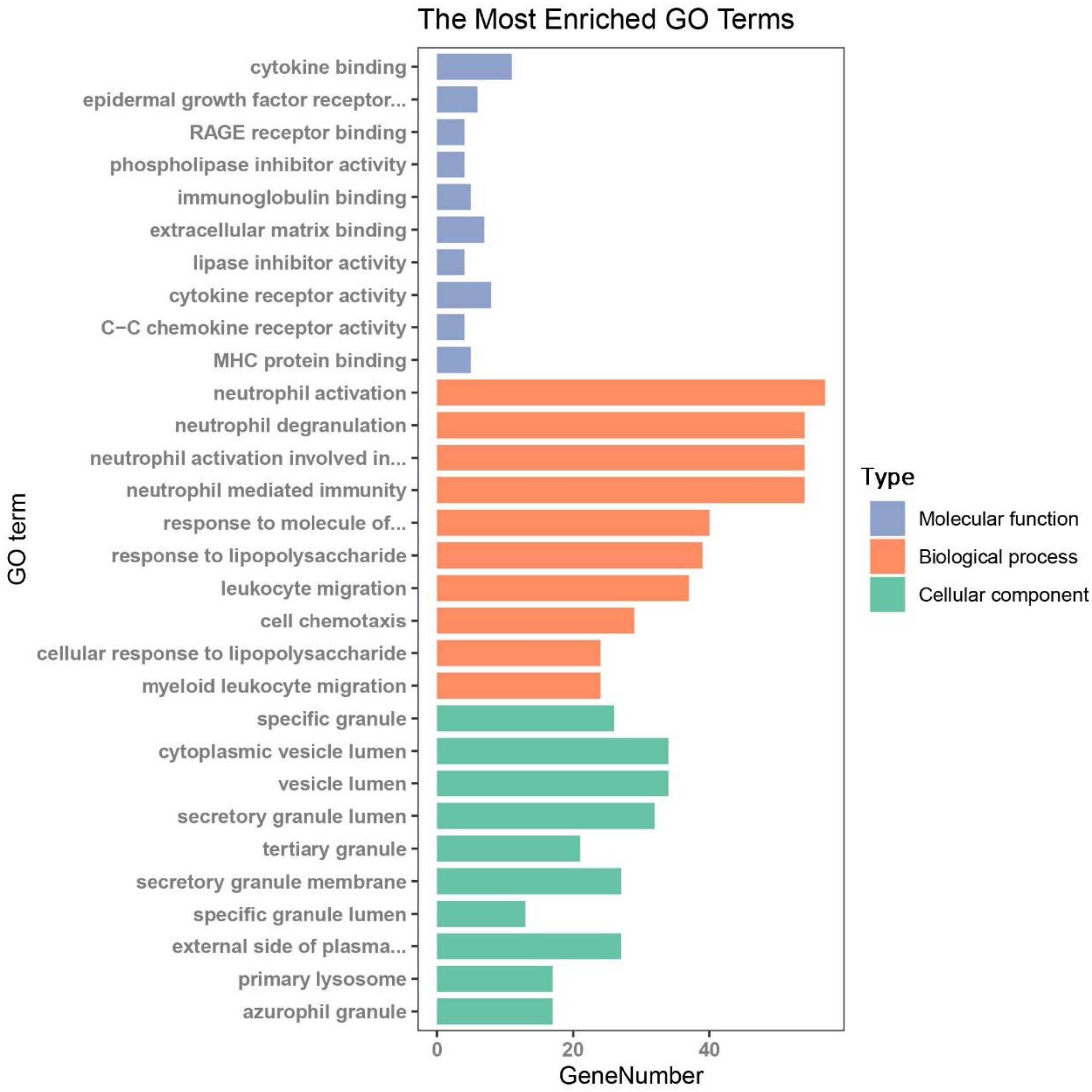

Figure S22. The GO enrichment analysis plot of the DEGs in EXP-CD4-HIV-Infection.

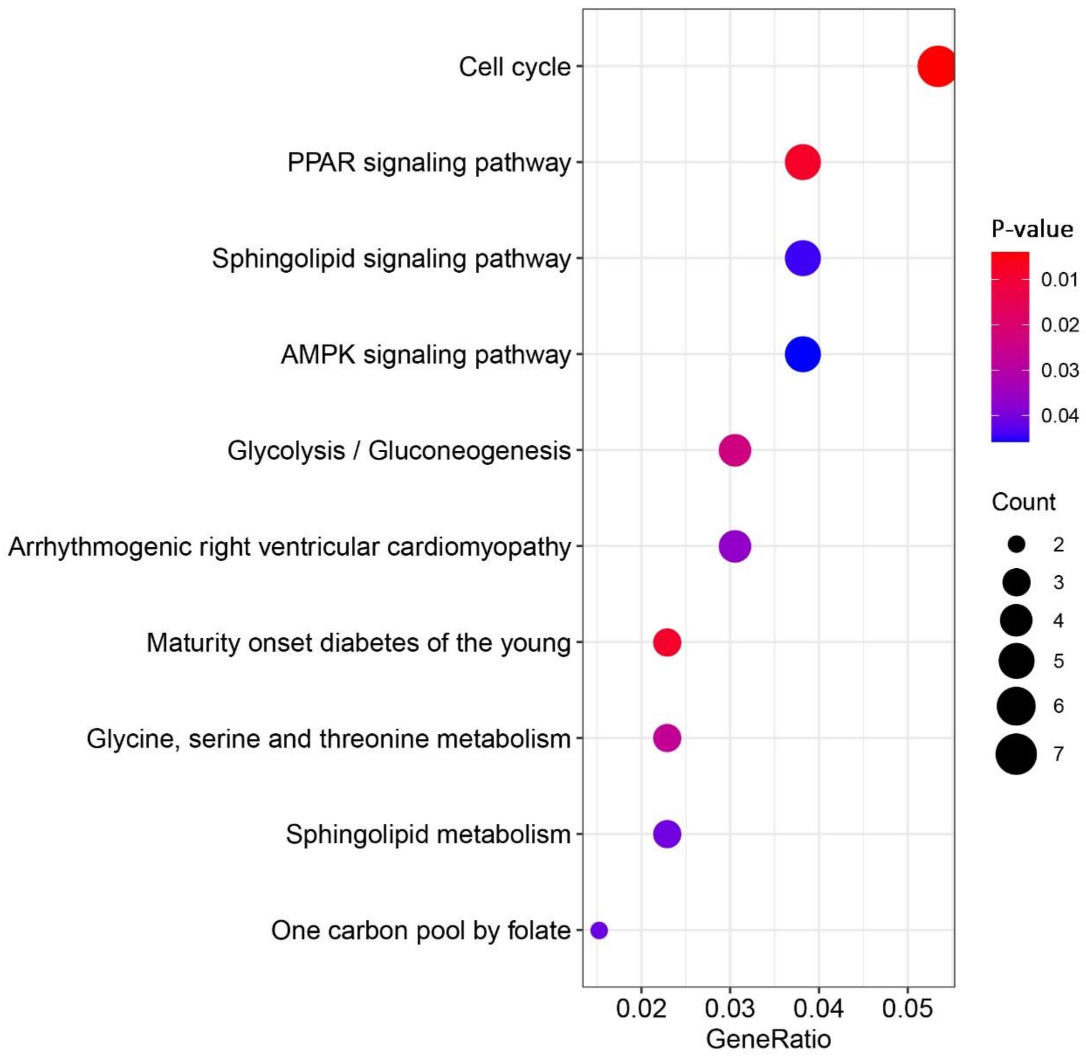

Figure S23. The pathway enrichment analysis plot of the DEGs in EXP-Blood-HIV-Infection.

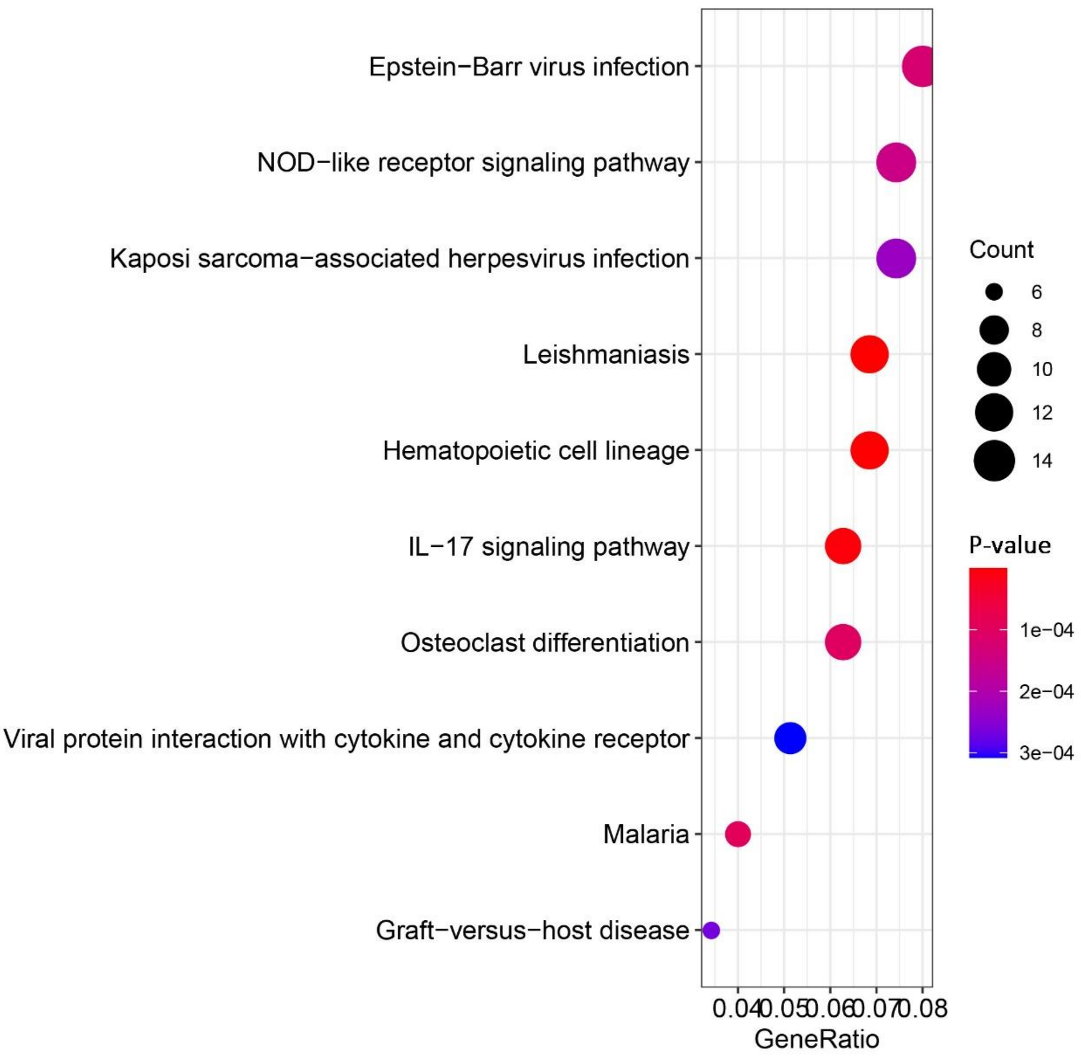

Figure S24. The pathway enrichment analysis plot of the DEGs in EXP-CD4-HIV-Infection.

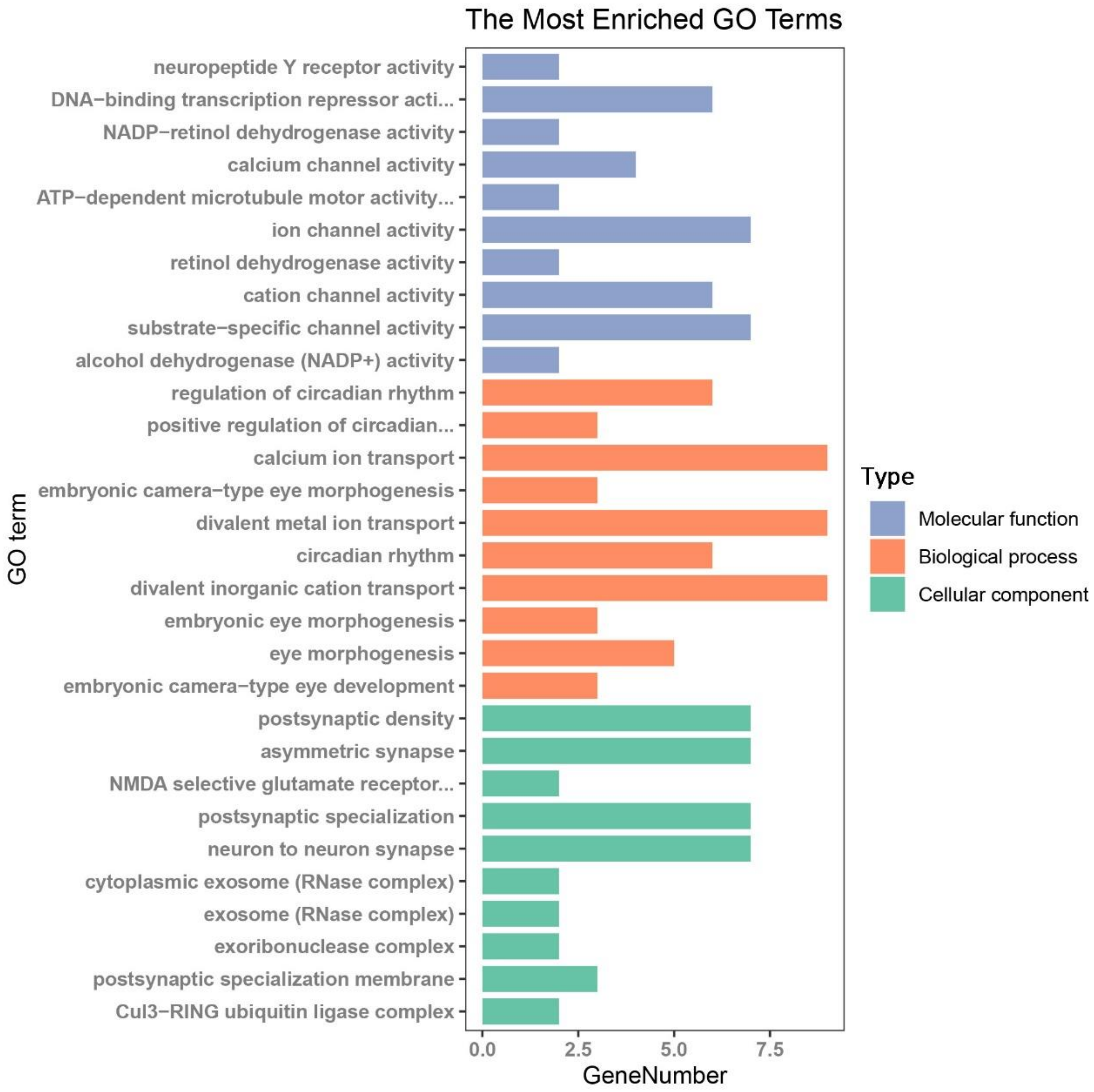

Figure S25. The GO enrichment analysis plot of the hyper-methylated down-regulated genes.

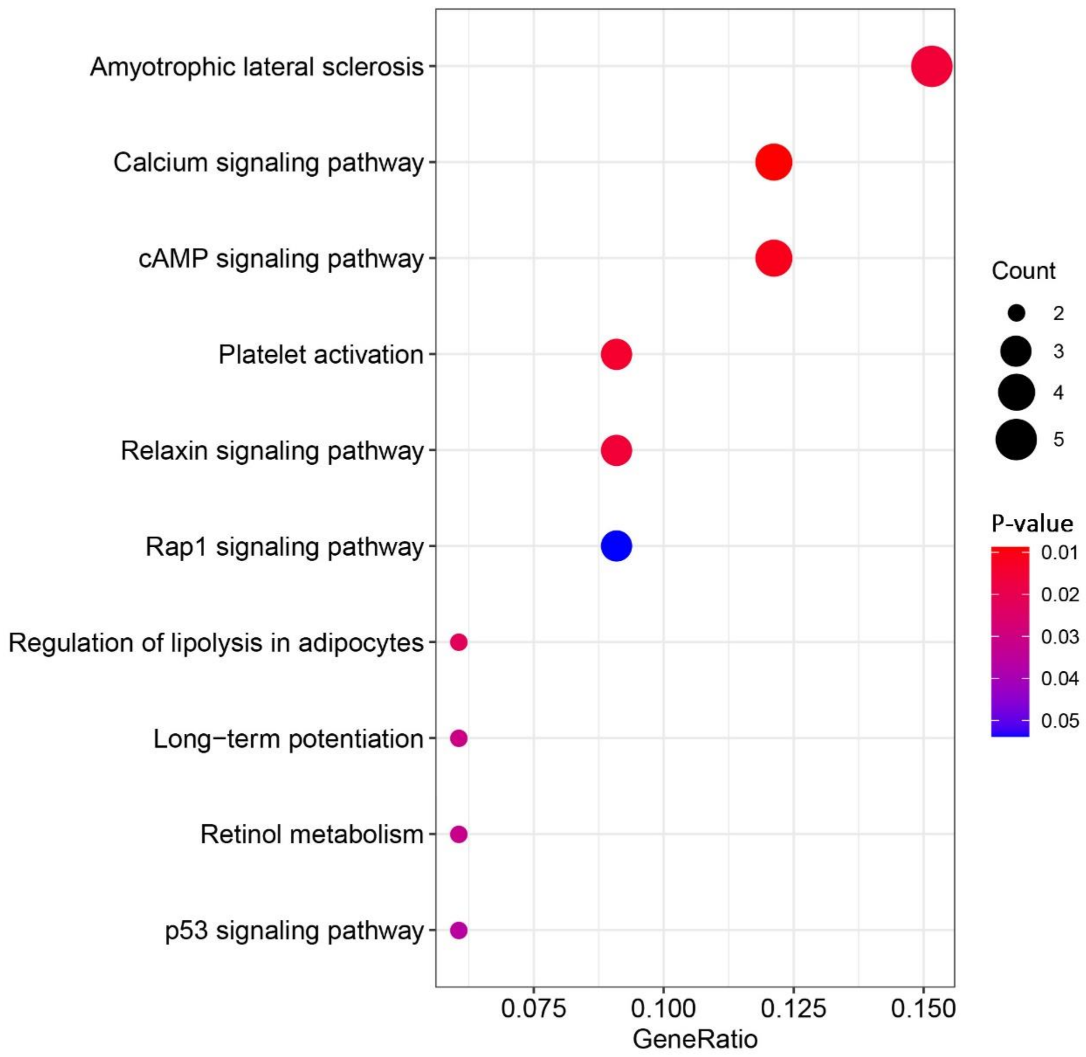

Figure S26. The pathway enrichment analysis plot of the hyper-methylated down-regulated genes.

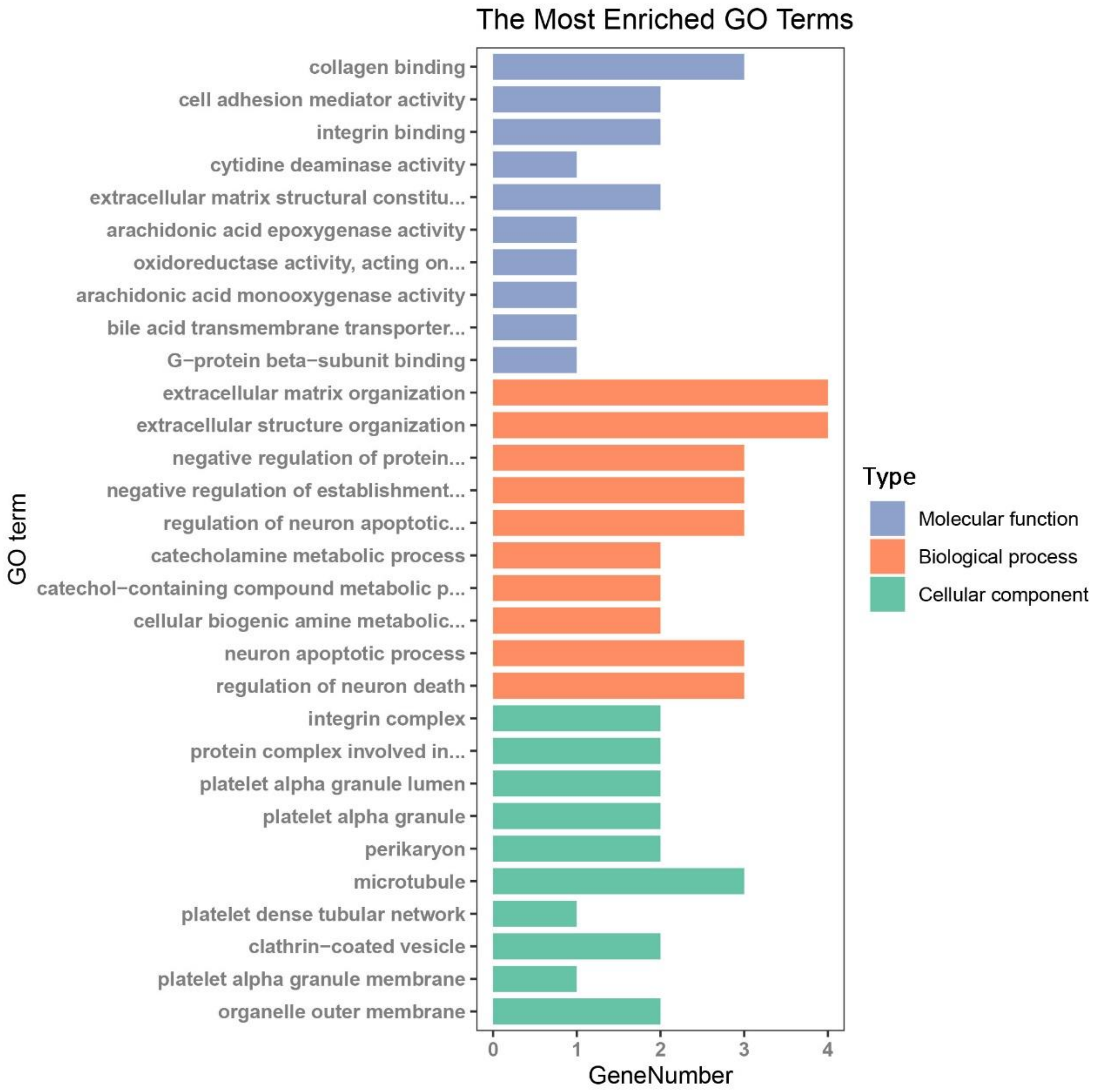

Figure S27. The GO enrichment analysis plot of the hypo-methylated up-regulated genes.

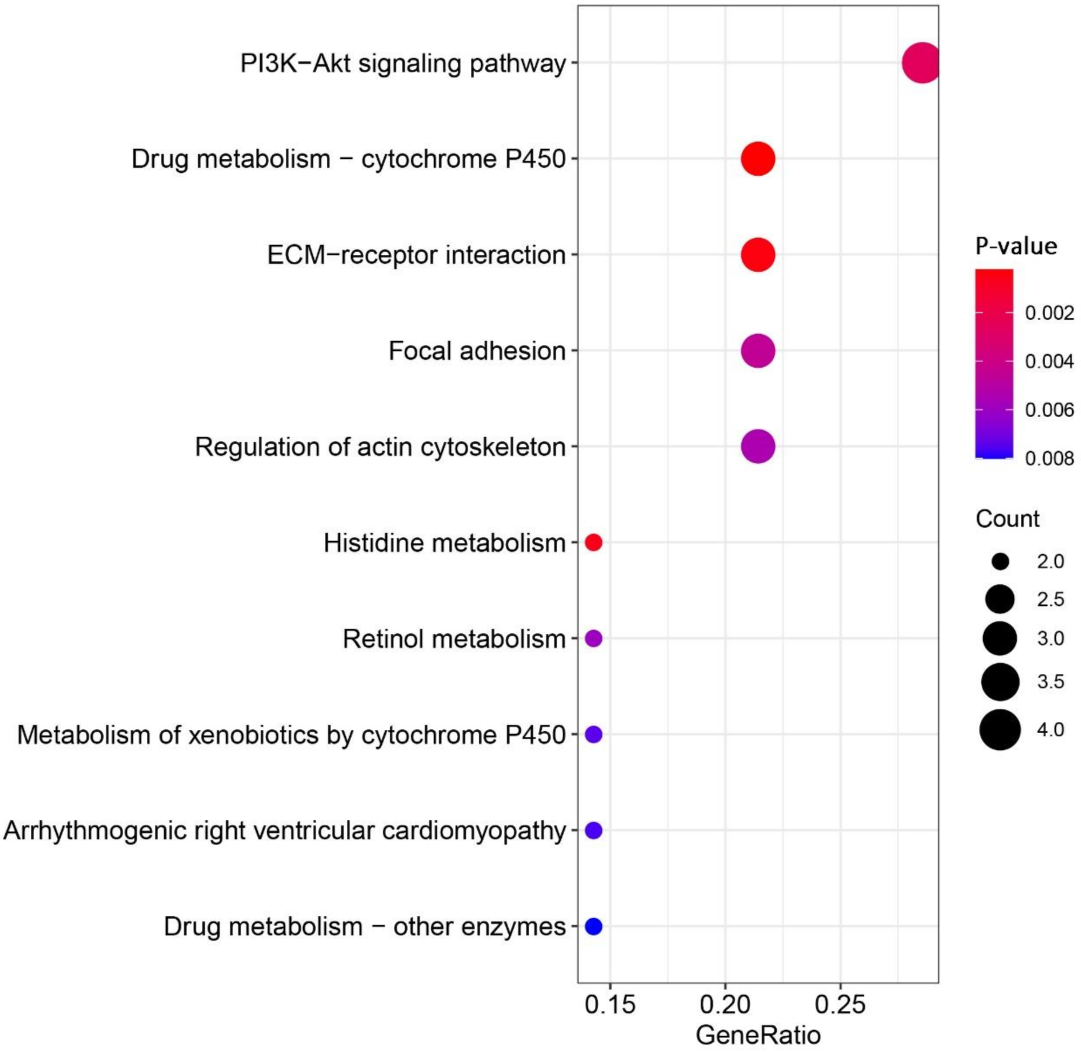

Figure S28. The pathway enrichment analysis plot of the hypo-methylated up-regulated genes.

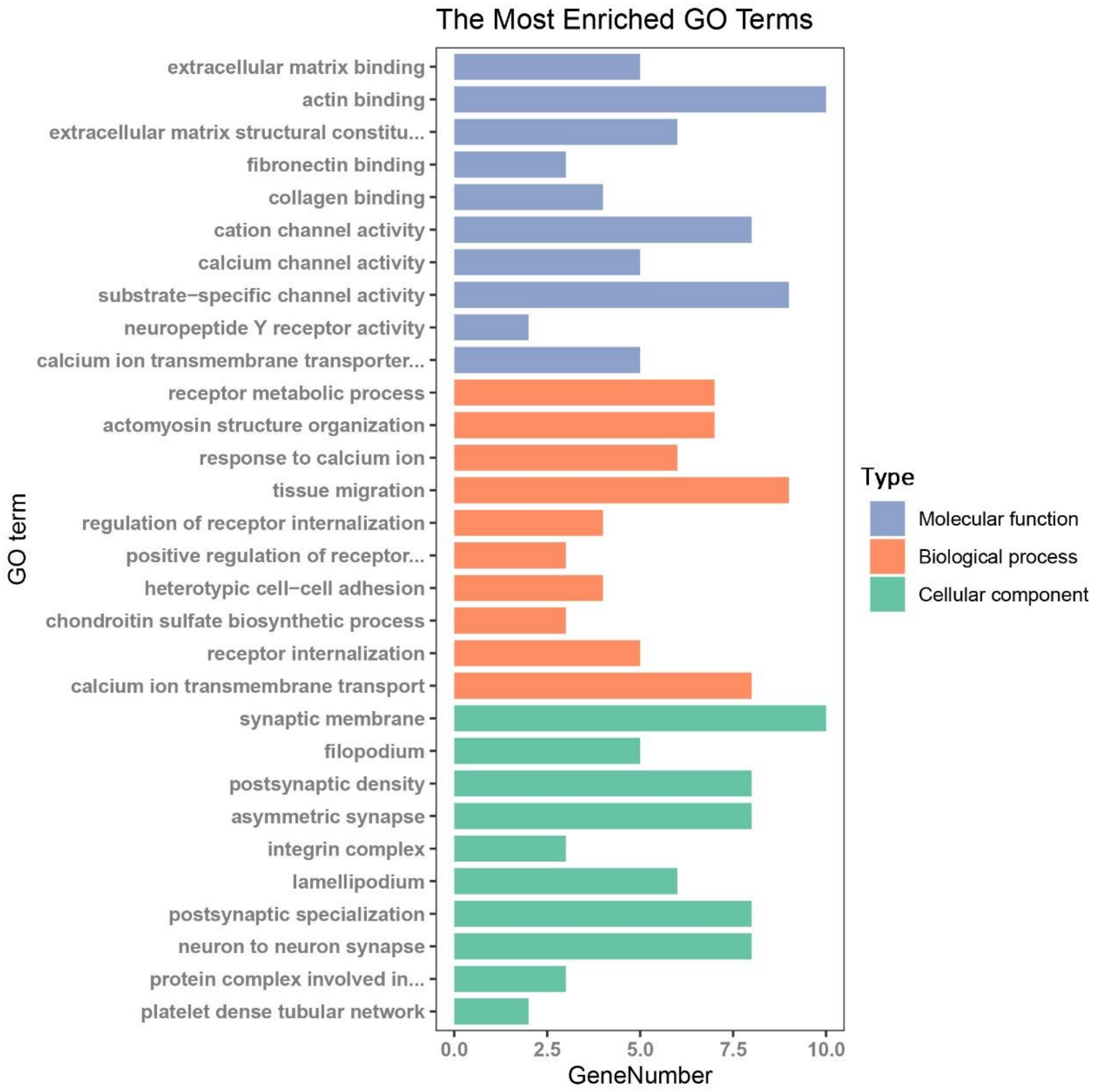

Figure S29. The GO enrichment analysis plot of the overlapped DEGs between EXP-Blood-HIV-Resistance and the ChIP-Seq data.

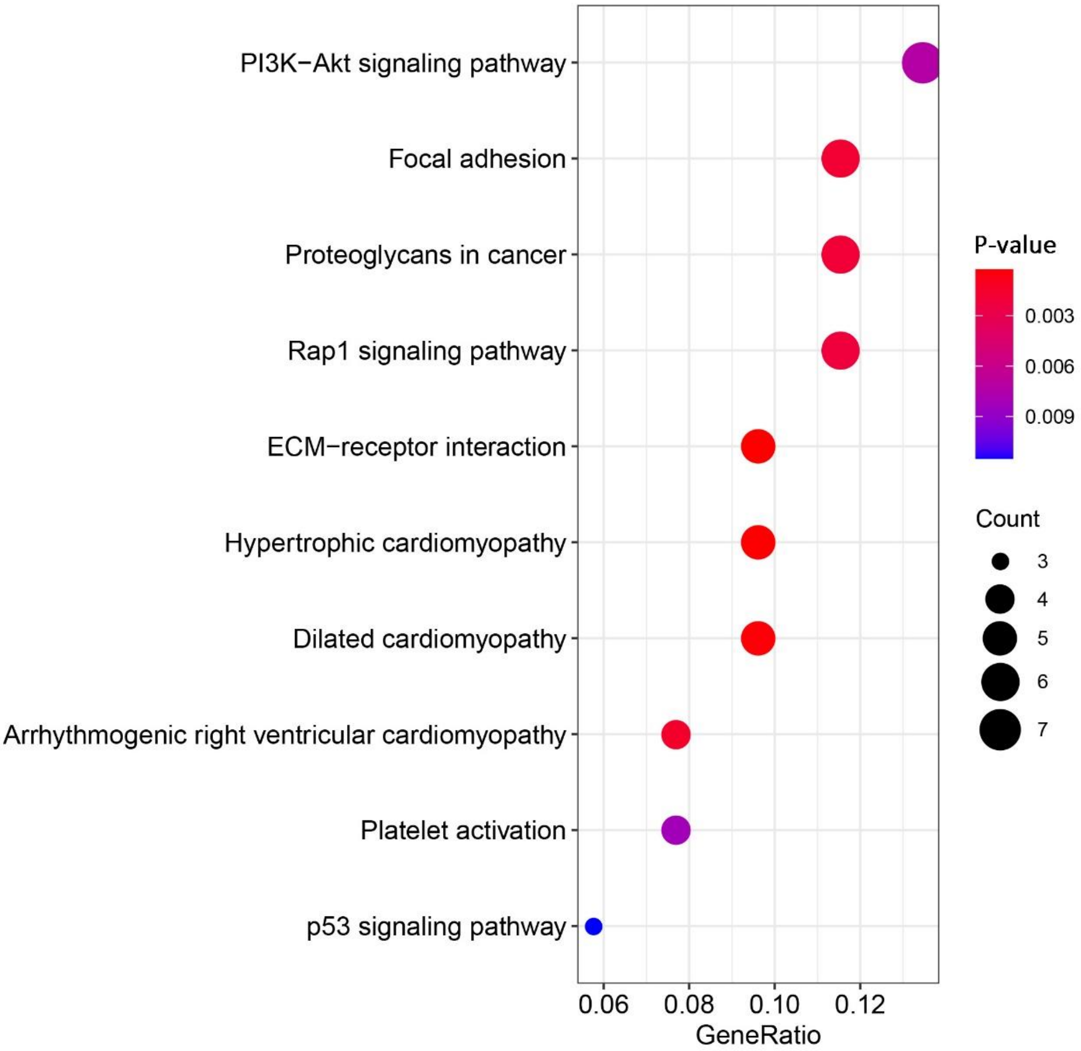

Figure S30. The pathway enrichment analysis plot of the overlapped DEGs between EXP-Blood-HIV-Resistance and the ChIP-Seq data.

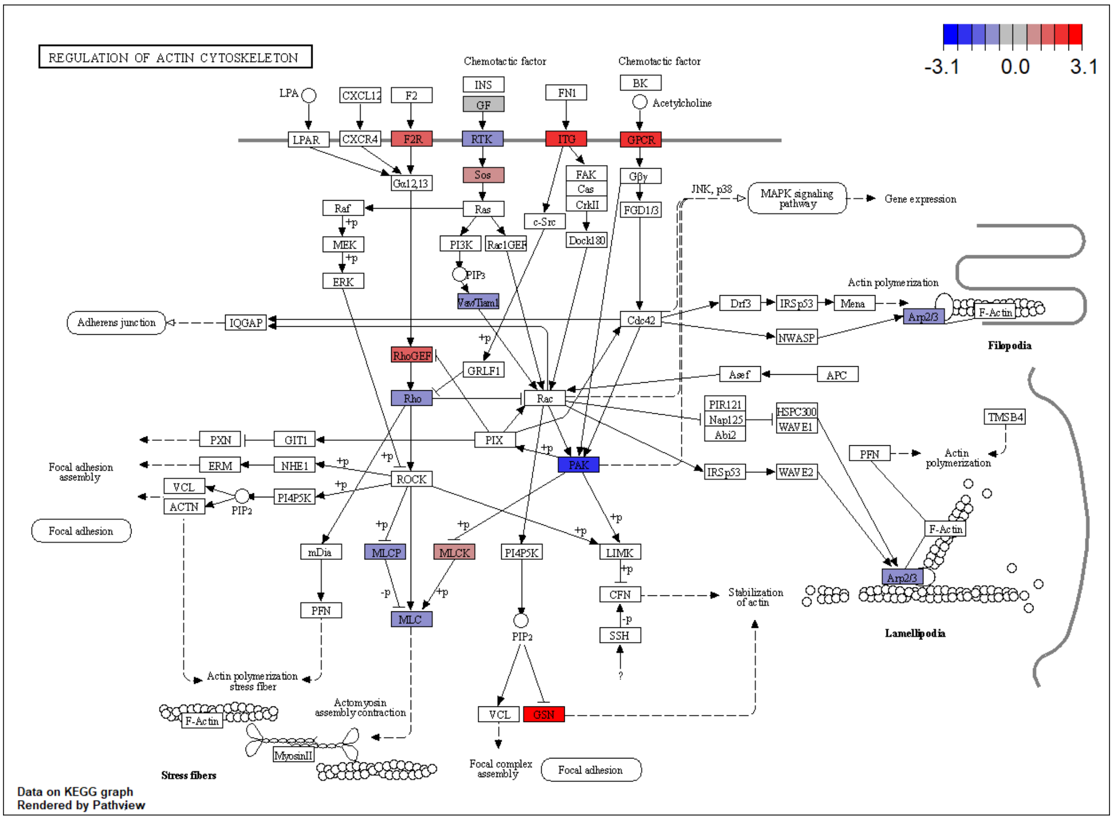

Figure S31. The enrichment of the DEGs in EXP-Blood-HIV-Resistance and EXP-CD4-HIV-Resistance in the pathway, regulation of actin cytoskeleton.

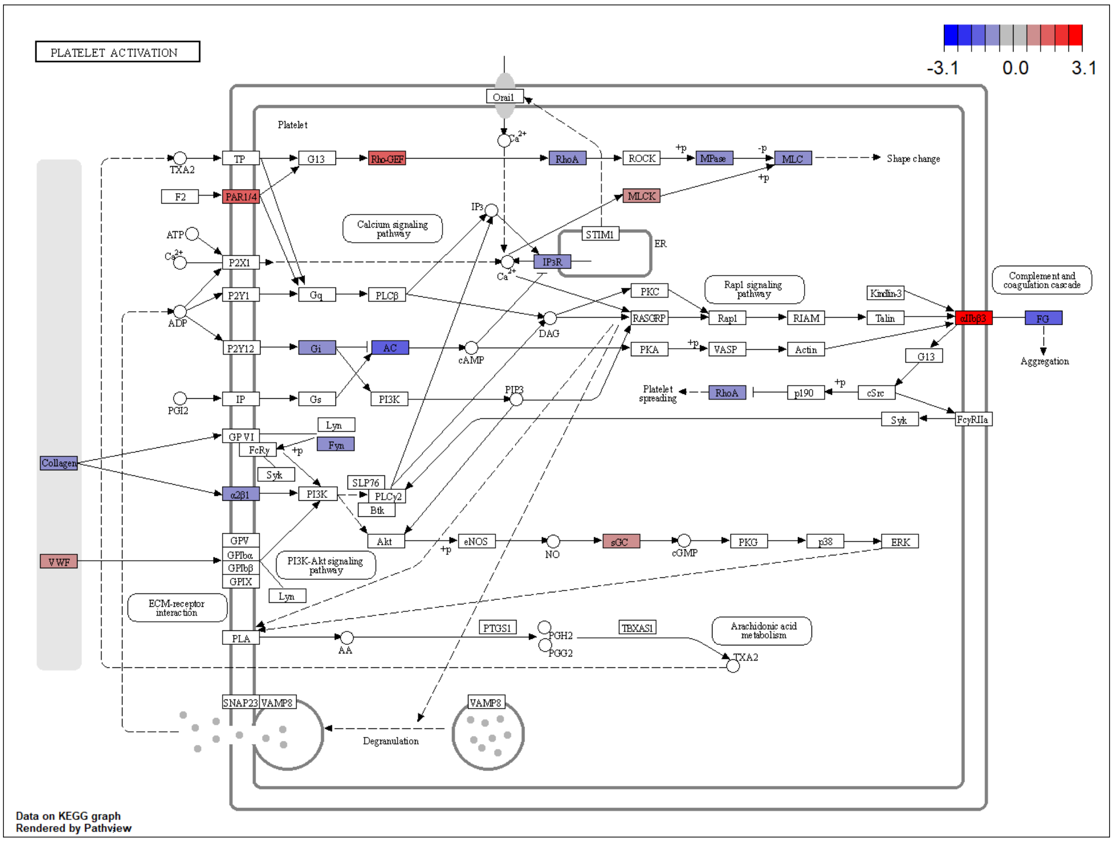

Figure S32. The enrichment of the DEGs in EXP-Blood-HIV-Resistance and EXP-CD4-HIV-Resistance in the pathway, platelet activation.

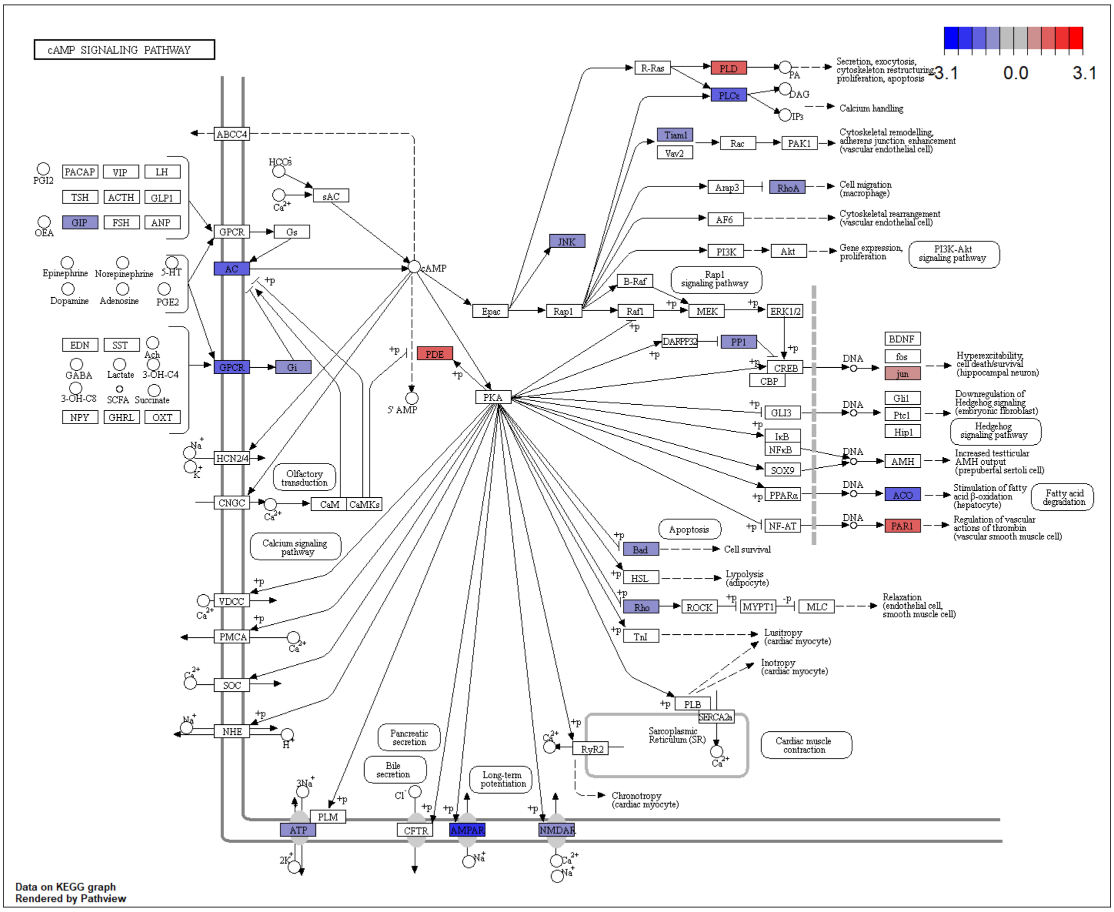

Figure S33. The enrichment of the DEGs in EXP-Blood-HIV-Resistance and EXP-CD4-HIV-Resistance in the pathway, cAMP signaling pathway.

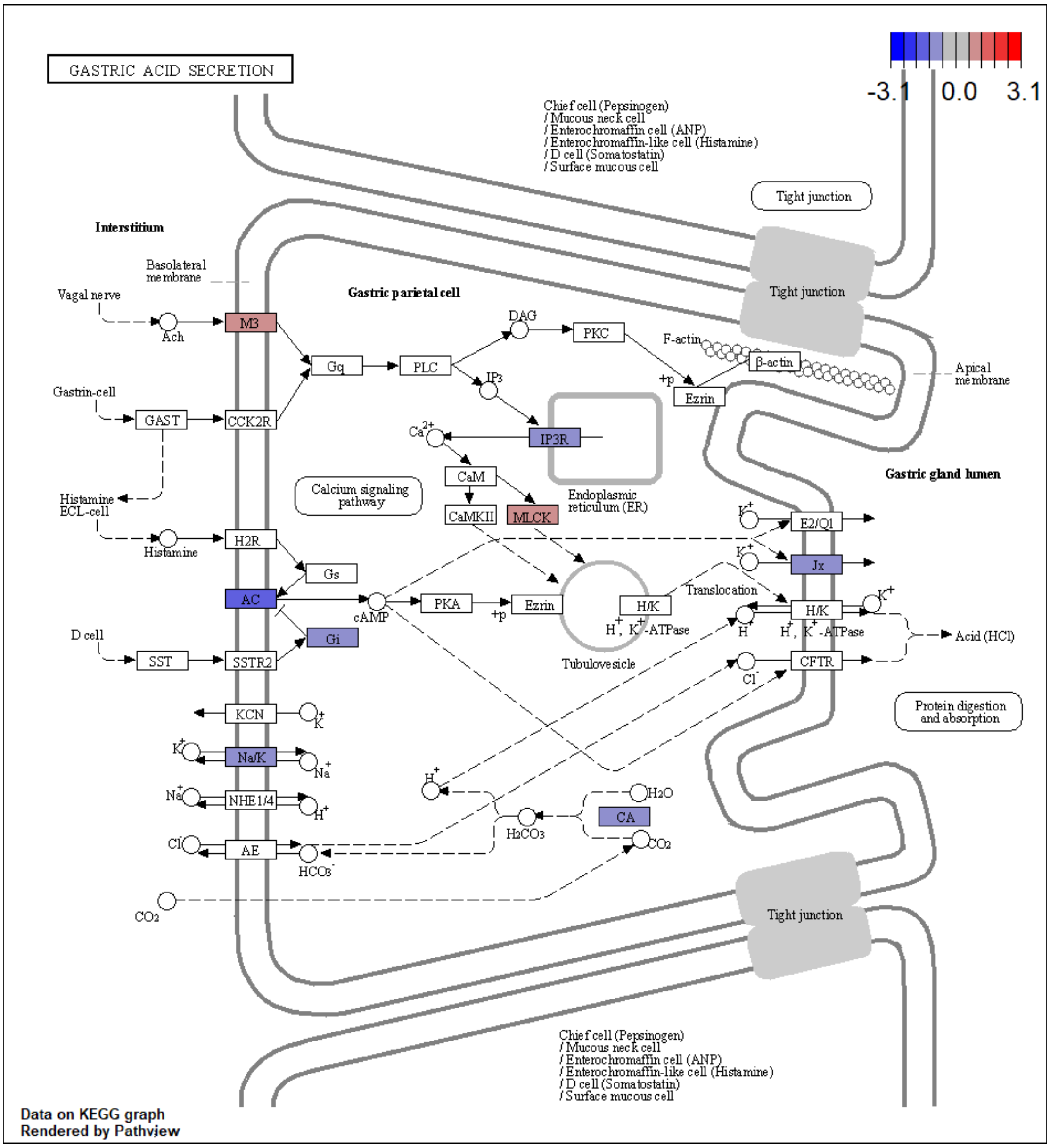

Figure S34. The enrichment of the DEGs in EXP-Blood-HIV-Resistance and EXP-CD4-HIV-Resistance in the pathway, gastric acid secretion.
